# Thioflavin T Inspirations: On the Photophysical and Aggregation Properties of Fluorescent Difluoroborates Based on the Benzothiazole Core

**DOI:** 10.1021/acs.jpca.5c01254

**Published:** 2025-04-09

**Authors:** Patryk Rybczyński, Agata Hajda, Robert Zaleśny, Borys Ośmiałowski, Joanna Olesiak-Bańska

**Affiliations:** † Faculty of Chemistry, Nicolaus Copernicus University, Gagarina Street 7, 87-100 Toruń, Poland; ‡ Faculty of Chemistry, Wroclaw University of Science and Technology, Wybrzeże Wyspiańskiego 27, 50-370 Wrocław, Poland

## Abstract

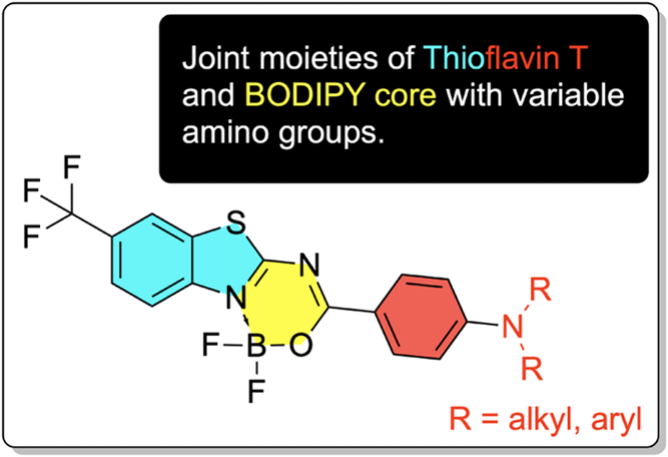

In this work, we investigated a novel series of fluorescent dyes based on benzothiazole core substituted with a palette of donor groups that differ in size, shape, and character (alkyl vs aromatic). These dyes exhibit an intramolecular charge transfer state in their electronic structure. The photophysical properties of the newly synthesized dyes were studied with an eye toward aggregation effects in solvents and mixtures of solvents differing in polarity. Moreover, multiphoton absorption studies were performed to determine the potential of the dyes in two-photon microscopy. The results of spectroscopic measurements were supported by electronic structure calculations using coupled-cluster theory. Overall, the results provide an indication regarding the optimal substituents to achieve bright emission in various media and how these properties are related to aggregation effects.

## Introduction

Fluorescent dyes demonstrate significant technological potential, driving researchers worldwide to synthesize new families of emitters followed by detailed spectroscopic characterization. Tunability of the properties of fluorescent dyes might be achieved by tweaking structural features. For example, dyes exhibiting aggregation-induced emission (AIE) usually carry flexible bulky groups that limit their rotational and vibrational degrees of freedom when aggregated.^1−3^ The restriction of intramolecular motions in aggregates results in increased fluorescence quantum yield (FQY) values. The AIE-based luminogens (AIEgens) attract wide attention due to their unique optical properties like high FQY in aqueous solution, photostability,^4^ red-shifted emission compared to monomeric emitters, and large Stokes shift. The opposite effect is commonly referred to as aggregation-caused quenching (ACQ), where emission decreases upon aggregation. ACQ may be obtained by choosing relatively large, flat aromatic cores that stack in aggregates or when the emitter associates with other molecules. Then, strong π–π intermolecular interactions in aggregate/crystal hamper the fluorescence. This feature is important, as a large group of small-sized organic probes suffer from the ACQ phenomenon at high concentrations in aqueous solution, limiting their performance in bioimaging. In this context, AIEgens are an interesting alternative; however, ACQ might also be useful in applications, e.g., in fluorescence imaging of specific biological structures.^5,6^ Although it is now well-recognized how to modify the structure of fluorescent dyes to make use of AIE or ACQ properties, some types of applications require multiparameter optimization. One of the examples is fluorescent dyes used in two-photon microscopy (2PM). In general, the molecular probes used in 2PM should exhibit high values of two-photon brightness σ_2,*B*_ (the product of FQY and two-photon absorption (2PA) cross section (σ_2_), commonly expressed in Göppert–Mayer (GM) units). For effective use in bioimaging, the σ_2,*B*_ value should exceed 50 GM.^7^ In that regard, AIEgens show promising application in 2PM, where they present high FQY, while the use of two-photon excitation warrants record depths for brain imaging.^8,9^ The challenge in the design of efficient two-photon-excited probes lies not only in the simultaneous optimization of FQY and σ_2_ but also in retaining the AIE/ACQ properties and matching the desired absorption/emission window (λ_abs_/λ_em_). As will be discussed in the following, the present study contributes to these efforts.

Combining donor and acceptor moieties with a conjugated spacer is an effective route to obtain molecules characterized by intramolecular charge transfer (ICT)—a feature allowing efficient two-photon absorption. At the same time, these structural motifs allow for simultaneous optimization of λ_abs_/λ_em_, FQY, and σ_2_.^10^ Heterocyclic cores are popular electron-accepting moieties occurring in the structure of fluorescent probes, and their properties may be further enhanced, e.g., the *aza* heterocycle can be converted into a quaternary salt, leading to an extremely strong, cation-based acceptor. The quaternary nitrogen atom is present in Thioflavin T^11,12^ and its derivatives,^13^ a gold standard in the detection of amyloid formation.^14,15^ The current study describes the heterocyclic core substituted with a −CF_3_ group that was studied previously by some of the present authors, and it was found that FQY was highly dependent on the simultaneous effect of two substituents incorporated into the fluorescent core in solution^16^ and in the aggregated state.^17^ Taken together, the previous findings indicate that the heterocyclic core is a promising platform for obtaining dyes for bioimaging applications with high sensitivity to the environment.

**Scheme 1:**
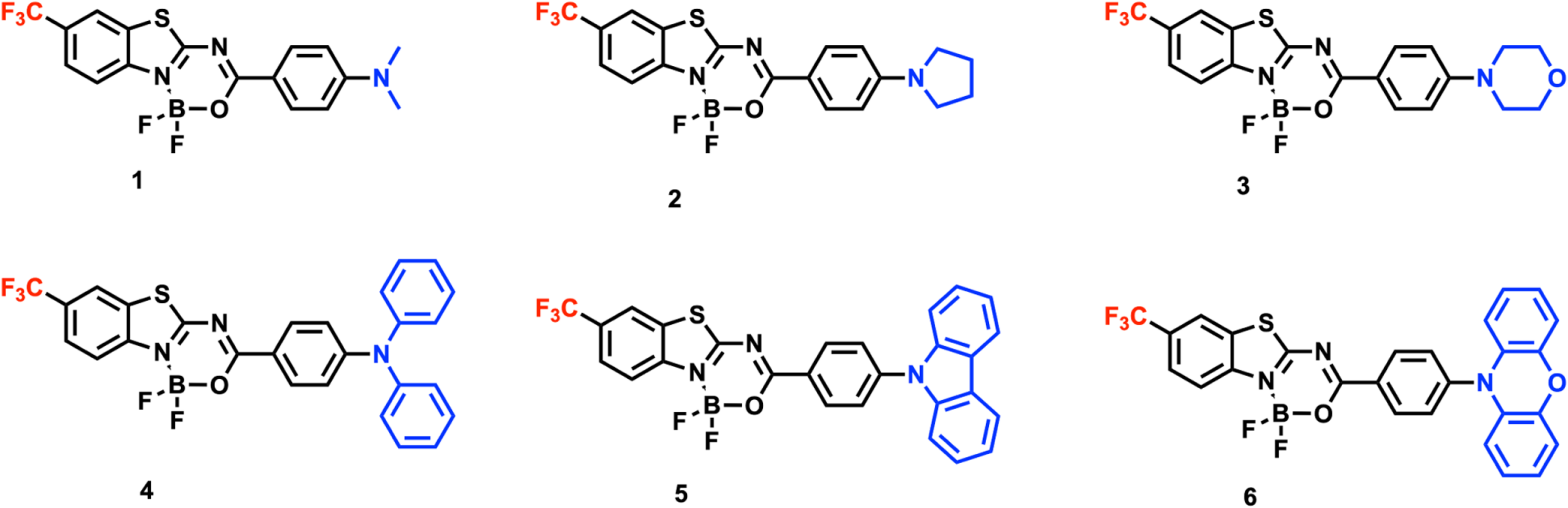
Structures of Studied Molecules

While the palette of donors is wide, the most common one is the *N,N*-dimethylamino group^18^ due to its various features, i.e., (a) it is prone to protonation,^10^ which results in a significant change in the position of bands in the spectra, and (b) it allows for tuning the π-conjugation in molecules due to structural changes in the geometry of the donating moiety.^19,20^ Thus, it comes as no surprise that the donating ability of the amino group is dependent on the type of group linked to the nitrogen atom. For example, Hammett substituent (*p*- substitution) constants for −NH_2_, −NHMe, and −NMe_2_ are −0.66, −0.70, and −0.83, respectively.^18^ Note that the Hammett substituent constant corresponding to the flexible *N,N*-diphenylamino group is equal to −0.22,^18^ and it falls in the range between –0.17 (−Me) and –0.27 (−OMe). It is important to remark that a rotational freedom of phenyls in the *N,N*-diphenylamino moiety is known to be responsible for the AIE phenomenon,^21−24^ while the presence of this electron-donating group safeguards substantial values of the 2PA cross section.^25^ Other aromatic amine-based donors, frequently used dyes exhibiting thermally activated delayed fluorescence (TADF),^26−29^ are phenoxazine and carbazole. Among the two substituents, the former was claimed to possess stronger electron-donating capabilities.^30^ A similar study comparing the donating ability of alkyl-substituted amino groups^31,32^ is also known. However, the direct comparison of alkyl-based versus aryl-based donors, sharing the same topology within the same fluorescent core, has not yet been thoroughly performed. In the present study, we will make an attempt to fill this gap by studying the photophysical properties of benzothiazole-based fluorescent dyes, as shown in Scheme 1, including the two-photon absorption phenomenon. These dyes combine the same electron-accepting moiety (CF_3_-substituted benzothiazole-BF_2_ core) and six electron-donating groups, categorized into alkyl-based donors (**1**: *N,N*-dimethylamine; **2**: pirrolidine; **3**: morpholine) and their aromatic counterparts (**4**: *N,N*-diphenylamine; **5**: carbazol; and **6**: phenoxazine). As shown previously, benzothiazole-based ICT dyes exhibit very promising two-photon absorption properties^33−35^ and were used in amyloid aggregation.^36^ With this in mind, the aim of the current work is to link the structure and type of electron-donating groups with (a) fluorescence quantum yield, (b) the position of absorption and emission band maxima, (c) AIE/ACQ properties, and (d) two-photon absorption cross section. To that end, we will employ a palette of experimental and computational techniques.

## Experimental and Computational Methods

### Synthesis and Characterization

The complexes were obtained by the reaction of amides (1 equiv) using BF_3_ · OEt_2_ (3 equiv) and DIPEA (2 equiv) as a base in anhydrous DCM adopting a procedure described earlier.^37^ Compounds **1**–**6** were purified by flash chromatography using a hexane–DCM gradient. The amides were obtained according to the literature description.^38−41^

#### Compound 1

Yield 34 mg (52%). Green powder, mp 285–286 °C. ^1^H NMR (700 MHz, from TMS, CDCl_3_): δ(ppm) 8.28 (d, 2H, ^3^*J* = 9.2 Hz), 8.05 (d, 1H, ^3^*J* = 8.5 Hz), 8.02 (s, 1H), 7.78 (dd, 1H, ^3^*J* = 8.5, ^4^*J* = 1.7 Hz), 6.77 (d, 2H, ^3^*J* = 9.2 Hz), 3.15 (s, 6H). ^11^B (128 MHz, from BF_3_ · Et_2_O, CDCl_3_): δ (ppm) 0.512 (t). ^13^C{^1^H} (100 MHz, from TMS, CDCl_3_) δ (ppm): 175.1, 169.1, 154.7, 142.7, 133.6, 128.2, 127.9, 126.9, 125.2, 122.4, 122.4, 119.6, 119.6, 118.1, 117.4, 111.6, 40.5. ^19^F (376 MHz, from CFCl_3_, CDCl_3_): δ (ppm) −62.8, −138.2. APCI (*m*/*z*) [M]^+^ 414.0878 cal. 414.0871 for C_17_H_13_BF_5_N_3_OS

#### Compound 2

Yield 166 mg (74%). Yellow powder, mp 286–287 °C. ^1^H NMR (400 MHz, from TMS, CDCl_3_): δ(ppm) 8.26 (d, 2H, ^3^*J* = 9.1 Hz), 8.04 (d, 1H, ^3^*J* = 8.6 Hz), 8.00 (s, 1H), 7.77 (dd, 1H, ^3^*J* = 8.6, ^4^*J* = 1.3 Hz), 6.58 (d, 2H, ^3^*J* = 9.2 Hz), 3.45 (s, 4H), 2.08 (s, 4H). ^11^B (128 MHz, from BF_3_ · Et_2_O, CDCl_3_): δ (ppm) 0.493 (t). ^13^C{^1^H} (100 MHz, from TMS, CDCl_3_) δ (ppm): 174.9, 169.0, 152.7, 142.5, 133.6, 127.7, 126.8, 125.0, 125.0, 124.9, 122.3, 119.5, 119.5, 117.9, 116.0, 111.5, 47.8, 25.4. ^19^F (376 MHz, from CFCl_3_, CDCl_3_): δ (ppm) −62.8, −138.5. APCI (*m*/*z*) [M]^+^ 440.1035 cal. 440.1027 for C_19_H_15_BF_5_N_3_OS

#### Compound 3

Yield 130 mg (66%). Green powder, mp >292 °C (dec.). ^1^H NMR (700 MHz, from TMS, CDCl_3_): δ(ppm) 8.30 (d, 2H, ^3^*J* = 9.2 Hz), 8.08 (d, 1H, ^3^*J* = 8.6 Hz), 8.04 (s, 1H), 7.78 (dd, 1H, ^3^*J* = 8.8, ^4^*J* = 1.2 Hz), 6.92 (d, 2H, ^3^*J* = 9.3 Hz), 3.88 (m, 4H) 3.43 (m, 4H). ^11^B (128 MHz, from BF_3_ · Et_2_O, CDCl_3_): δ (ppm) 0.516 (t). ^13^C{^1^H} (100 MHz, from TMS, CDCl_3_) δ (ppm): 175.3, 168.8, 155.4, 142.6, 133.3, 128.4, 128.1, 127.8, 127.0, 125.2, 124.9, 122.2, 119.68, 119.6, 119.6, 118.3, 113.1, 66.4, 47.1. ^19^F (376 MHz, from CFCl_3_, CDCl_3_): δ (ppm) −62.8, −137.7. APCI (*m*/*z*) [M]^+^ 456.09983 cal. 456.0976 for C_19_H_15_BF_5_N_3_O_2_S

#### Compound 4

Yield 19 mg (37%). Yellow powder, mp 201–202 °C. ^1^H NMR (700 MHz, from TMS, CDCl_3_): δ(ppm) 8.20 (d, 2H, ^3^*J* = 9.1 Hz), 8.08 (d, 1H, ^3^*J* = 8.6 Hz), 8.05 (s, 1H), 7.78 (dd, 1H, ^3^*J* = 8.7, ^4^*J* = 1.2 Hz), 7.37 (m, 4H) 7.21 (m, 6H) 7.79 (d, 2H, ^3^*J* = 9.1 Hz). ^11^B (128 MHz, from BF_3_ · Et_2_O, CDCl_3_): δ (ppm) 0.533 (t). ^13^C{^1^H} (100 MHz, from TMS, CDCl_3_) δ (ppm): 175.3, 168.7, 154.1, 145.8, 142.6, 132.7, 129.8, 129.5, 128.5, 128.1, 127.1, 126.6, 125.6, 125.2, 125.2, 124.9, 122.2, 121.1, 119.7, 119.6, 118.7, 118.4. ^19^F (376 MHz, from CFCl_3_, CDCl_3_): δ (ppm) −62.8, −137.6. APCI (*m*/*z*) [M]^+^ 538.1193 cal. 538.1184 for C_27_H_17_BF_5_N_3_OS

#### Compound 5

Yield 15 mg (30%). Green powder, mp >236 °C (dec.) ^1^H NMR (700 MHz, from TMS, CDCl_3_): δ(ppm) 8.66 (d, 2H, ^3^*J* = 8.7 Hz), 8.19 (d, 1H, ^3^*J* = 8.8 Hz), 8.02 (m, 3H), 7.89 (dd, 1H, ^3^*J* = 8.7, ^4^*J* = 1.3 Hz), 7.81 (d, 2H, ^3^*J* = 8.7 Hz), 7.55 (d, 2H, ^3^*J* = 8.1 Hz), 7.46 (m, 2H) 7.34 (m, 2H). ^11^B (128 MHz, from BF_3_ · Et_2_O, CDCl_3_): δ (ppm) 0.682 (t). ^13^C{^1^H} (100 MHz, from TMS, CDCl_3_) δ (ppm): 175.6, 168.5, 144.1, 140.0, 132.6, 126.4, 125.6, 124.1, 121.0, 120.6, 119.9, 119.0, 109.9. ^19^F (376 MHz, from CFCl_3_, CDCl_3_): δ (ppm) −62.9, −136.3. APCI (*m*/*z*) [M]^+^ 536.1034 cal. 536.1027 for C_27_H_15_BF_5_N_3_OS

#### Compound 6

Yield 58 mg (53%). Purple powder, mp 251–253 °C. ^1^H NMR (700 MHz, from TMS, CDCl_3_): δ(ppm) 8.63 (d, 2H, ^3^*J* = 8.8 Hz), 8.19 (d, 1H, ^3^*J* = 8.7 Hz), 8.15 (s, 1H), 7.89 (dd, 1H, ^3^*J* = 8.8, ^4^*J* = 1.5 Hz), 7.56 (d, 2H, ^3^*J* = 8.7 Hz), 6.73 (m, 4H), 6.64 (m, 2H), 6.04 (dd, 1H, ^3^*J* = 7.8, ^4^*J* = 1.3 Hz). ^11^B (128 MHz, from BF_3_ · Et_2_O, CDCl_3_): δ (ppm) 0.677 (t). ^13^C{^1^H} (100 MHz, from TMS, CDCl_3_) δ (ppm): 175.6, 168.4, 145.7, 144.3, 133.4, 133.4, 130.9, 130.8, 130.2, 129.3, 128.9, 127.5, 125.7, 123.4, 122.2, 120.0, 119.9, 119.1, 115.9, 113.7. ^19^F (376 MHz, from CFCl_3_, CDCl_3_): δ (ppm) −62.9, −136.2. APCI (*m*/*z*) [M]^+^ 552.0983 cal. 552.0976 for C_27_H_15_BF_5_N_3_O_2_S

### Photophysical Measurements

Chloroform was used as a solvent to warrant that aggregation was not present during photophysical measurements. It is safe to assume that aggregation in chloroform was not present because the ^13^C NMR spectra were recorded in CDCl_3_ without any traces of precipitation. The absorption spectra were recorded by using a Shimadzu UV-1900 spectrophotometer. The photoluminescence spectra, FQYs (using integrating sphere), and fluorescence decays (using time-correlated single photon counting, Figure S29) were measured by using an FS5 spectrometer (Edinburgh Instruments). Lifetimes were calculated using FAST software. Nonlinear optical measurements and calculation of σ_2_: two-photon excited fluorescence (2PEF) spectra were measured using a custom-built multiphoton setup. The excitation source was a femtosecond mode-locked Ti:sapphire laser (∼100 fs, 80 MHz, Chameleon, Coherent Inc.) with an operating wavelength range of 680–1080 nm. The emission spectra were measured with a spectrograph—Shamrock 303i (Andor) with an iDUS camera (Andor). Optical filters were also used: FELH0700—Ø25.0 mm Longpass Filter (Thorlabs) and FESH0700 (Thorlabs). The sample and reference dye were always measured at the same excitation power (20 mW). 2PA cross sections (σ_2_) were calculated with the following equation^42^
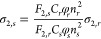
where *r* and *s* denote the reference and sample, respectively. φ_*r*_ and φ_*s*_ are the fluorescence quantum yields, *F*_2*,s*_ and *F*_2*,r*_ are the integrated two-photon fluorescence intensity at a particular excitation wavelength, and *n* is the refractive index of the solvent. *C*_*s*_ and *C*_*r*_ are the concentrations of the sample and reference, respectively. Fluorescein in 0.1 M NaOH was used as a reference (FQY equal to 0.95 taken from the literature^43^). The value of σ_2_ for fluorescein was obtained from a previous report.^42^ Addressing 2PEF of dyes **4** and **5**: It was not possible to collect the full spectrum (due to the cut emission by emission filters), and the tail of the spectrum above 650 nm was fitted in Origin Pro based on 1PEF and added to integrated two-photon fluorescence intensity to obtain the value of the integral of the full spectrum. In order to confirm the two-photon nature of the observed fluorescence excited by laser pulses, we measured fluorescence intensity versus incident laser excitation power and determined the power exponent (*n*). For each power, two 2PEF spectra were collected to observe if photobleaching did not take place upon increasing laser power. The power exponent was calculated using the following equation
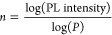
where PL intensity is the 2PEF intensity and *P* is the average incident laser power.

### Computational Details

The studied dyes were solvated with chloroform molecules, resulting in spherically symmetric clusters.^44^ Two-layer ONIOM calculations were performed for the clusters.^44^ The initial composition of layers was determined based on the following criteria
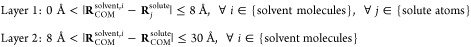
where **R**_COM_^solvent,*i*^ refers to the position vector of the center of mass (COM) of the *i*th solvent molecule and **R**_*j*_^solute^ refers to the *j*th atom of the solute. **R**_COM_^solute^ represents the position vector of COM of the solute molecule. Subsequently, the geometry optimization was performed using the GAUSSIAN program.^45^ In so doing, layer 1 was described at the B3LYP/6–31+G(d) level of theory with the D3 version of Grimme’s dispersion model,^46^ while layer 2 was described by the AMBER force field.^47^ The optimized clusters were subsequently used in electronic structure calculations to determine one- and two-photon absorption spectra. These calculations were performed using the TURBOMOLE program^48,49^ at the RI-CC2/aug-cc-pVDZ level of theory,^50^ and all solvent molecules were represented by point charges. The two-photon absorption cross section was computed assuming Gaussian broadening, and the corresponding expressions can be found elsewhere.^51^ In order to link the two-photon absorption activity with electronic structure parameters, we employed a generalized-few state model developed for non-Hermitian theories to decompose two-photon transition strength δ.^52,53^
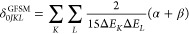
1Here,
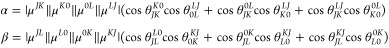
In the above expression, the superscripts distinguish between right (*L0*) and left (*0L*) moments and Δ*E*_*K*_ = 0.5 ω_*J*_–ω_*K*_. The term θ_*L*0_^*PQ*^ represents the angle between the transition dipole moment vectors μ^*PQ*^ and μ^*RS*^.

## Results and Discussion

As mentioned above, we study AIE/ACQ in a series of fluorescent compounds with variable donors (the acceptor part is kept intact). These dyes are prone to ICT upon electronic excitation, and our focus will be on such electronic states (usually the lowest-energy electronic singlet state). The most probable structural changes associated with geometrical variability (e.g., rotation or bending) are introduced in the electron-donating moiety, namely, at the C–N bond between 1,4-phenylene and donating nitrogen. Similar geometrical variability is possible at the bond joining the same nitrogen and side phenyls (for **4**). In two remaining aromatic substituents (carbazole **5** and phenoxazine **6**), both aromatic rings are bridged to disable rotations within the donor.

**Figure 1:**
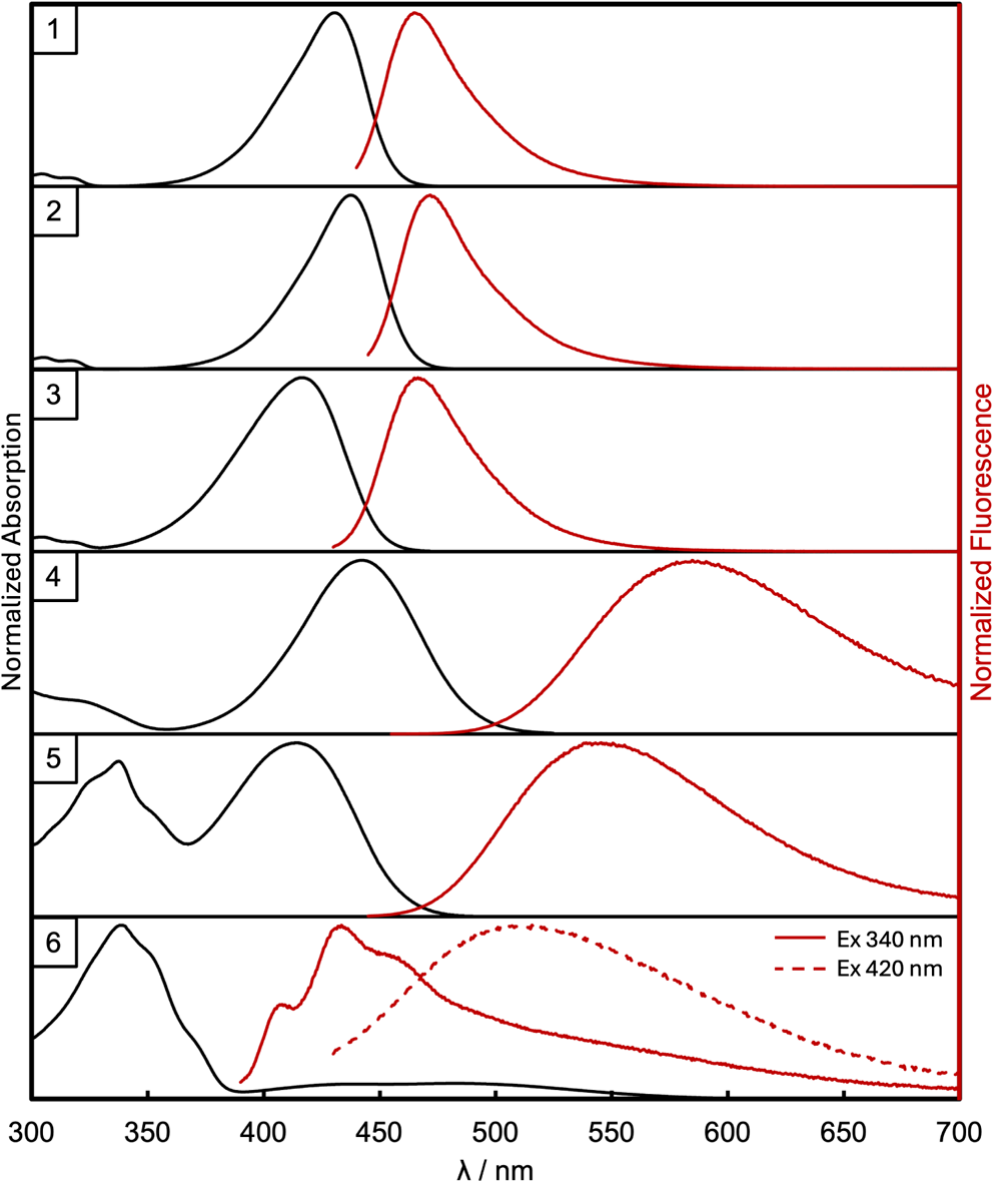
Normalized absorption (black) and fluorescence (red) spectra for **1**–**6** in chloroform. Excitation wavelengths (nm): 430 for **1**, 437 for **2**, 416 for **3**, 442 for **4**, 420 for **5**, and 340 and 420 for **6**. Concentration ca. 10^–5^ M for absorption and 10^–6^ M for emission.

The absorption and emission spectra for **1**–**6** are shown in Figure 1, while the numeric data are collected in Table 1. It is worth pointing out that for **6**, two sets of data (absorption and emission) are given, coming from the local (phenoxazine) and charge transfer features. However, for that compound, the dual character of emission is reported and refers to excitation at 380 (solid red line) and 420 nm (dashed red line, Figure 1). Moreover, since dye **6** is considered nonemissive in chloroform, it will be excluded from further studies in a more polar environment.

**Table 1:** Photophysical Properties for **1**–**6** in Chloroform^a^

	λ_abs_	ϵ	λ_em_	Stokes shift	FQY	τ	*k*_r_	*k*_nr_
comp.	[nm]	M^–1^ cm^–1^	[nm]	[cm^–1^]		[ns]	[10^7^ s^–1^]	[10^7^ s^–1^]
**1**	430	86,500	465	1723	0.94	1.80	52.4	3.34
**2**	437	98,800	472	1648	0.95	1.83	52.3	2.75
**3**	416	78,100	466	2516	0.98	1.73	56.6	1.16
**4**	442	55,700	585	5530	0.63	3.94	16.0	9.39
**5**	414	28,000	542	5852	0.58	6.32	9.17	6.64
**6**	338/484	43,900	433/515	6491/1244	>0.01	1.66	0.60	59.5

a Absorption maxima reported for low energy bands (ICT state), except for **6**, where two values are given.

Compound **1** was known previously^38^ as a member of a series of donor–acceptor molecules substituted in the benzothiazole ring. While the maximum of absorption for **1** in toluene was at 428 nm and FQY is equal to 0.93 (at λ_em_ = 460 nm), similar skeletons look attractive for two-photon absorption-based bioapplications. In fact, one can expect a decrease in FQY in more polar solvents, as in the case of **1** in acetonitrile (FQY = 0.05, λ_em_ = 500 nm, negligible change of absorption), but the dye may still be useful in 2PM as long as σ_2,*B*_ is above 50 GM. The comparison of the alkyl-based subset of dyes **1**–**3**, dissolved in chloroform, reveals slight differences in the positions of λ_abs_ and λ_em_. Derivatives **4** and **5** exhibit significantly broader absorption and emission bands in contrast to dyes **1**–**3**. Compound **5** has the largest Stokes shift in the series **1**–**5**, amounting to 5852 cm^–1^. On the other hand, the emission maximum of compound **4** is the most bathochromically shifted compared to the other compounds, reaching the yellow part of the spectrum (585 nm). The change in the donor structure on passing from compounds **1** to **3** has little effect on the FQY values. The same applies to fluorescence lifetimes (τ) and the radiative (*k*_r_ = FQY/τ) and nonradiative (*k*_nr_ = (1 – FQY)/τ) rate constants. Taking into account the high FQY and τ of approximately 1.8 ns, the *k*_r_ ranges from 52× 10^7^ to 56× 10^7^ s^–1^, which is an order of magnitude higher than *k*_nr_.

**Figure 2:**
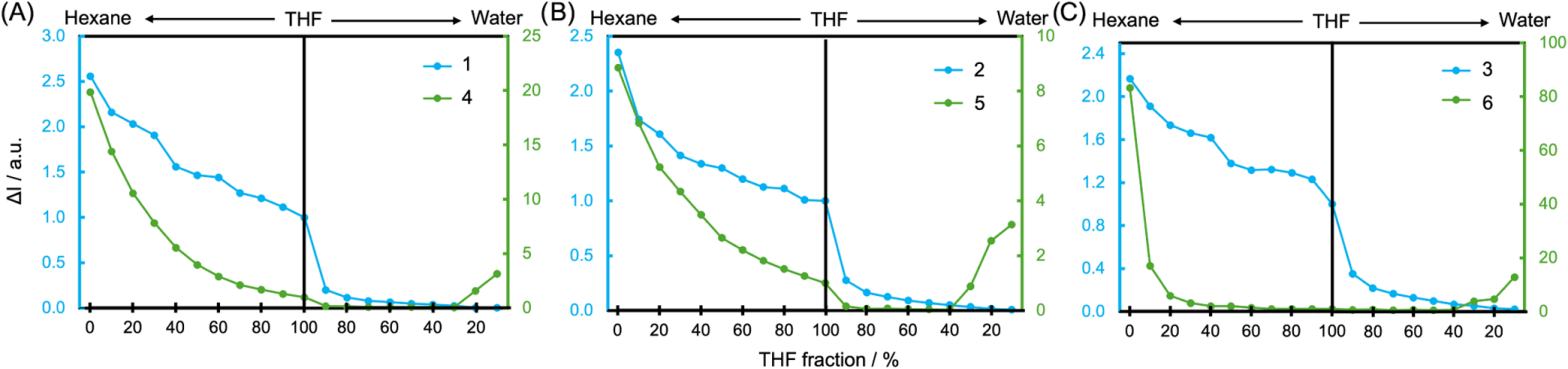
Change in emission intensity (normalized to 1 for THF at band maximum) for compounds **1**–**3** (in blue) and **4**–**6** (in green) depending on the composition of hexane/THF (or THF/water) mixtures.

The aggregation of molecules, except for an obvious concentration increase, occurs in a hydrophobic or hydrophilic environment. Understanding AIE/ACQ processes is essential for utilizing molecules in diverse environments or detecting specific biomolecules. This is especially important for dye interactions in local pockets in biomaterials, which can be hydrophobic or hydrophilic. In that regard, the response of dyes can be studied using mixtures of appropriate solvents, which mimic the target environment. Since the studied molecules are intended for future use in biolabeling, the sensitivity of emission parameters to the polarity change was evaluated in hexane/THF mixtures and also to inspect AIE or ACQ phenomena in THF/water mixtures. As the polarity changes (hexane/THF, data in Table 2), the position of the emission maximum shifts, which is typical for ICT molecules. In the case of alkyl derivatives **1**–**3**, the position of the emission band is very similar, so is the FQY. For **4** and **5**, the red shift is two times larger than for alkyl derivatives, while the emission drop is much steeper in **4** and **5** than that for alkyl derivatives. In the case of dye **6**, the emission is almost totally quenched at the initial course of the hexane/THF experiment (Figure 2). Figure S25 illustrates how the emission position varies depending on the composition of the hexane/THF mixture. It should be noted that τ (Table S1 in the SI) and FQY for derivatives **1**–**3** did not change significantly upon changing hexane to THF, so *k*_r_ and *k*_nr_ are also similar, while in the remaining derivatives, the contribution of nonradiative processes is dominant over radiative ones, producing dimmed emission.

**Table 2:** Maximum of Emission and Fluorescence Quantum Yields for **1**–**6**, Measured in Pure Solvents (Hexane and THF) and for Their Aggregates in a THF/Water Mixture (90% Water v/v)

	λ_em_	λ_em_	λ_em_	FQY	FQY	FQY
comp.	(hexane)	(THF)	(THF/H_2_O)	(hexane)	(THF)	(THF/H_2_O)
**1**	432.0	485.0	532.5	0.95	0.83	0.02
**2**	436.5	489.0	547.0	0.99	1.00	0.01
**3**	435.0	490.0	505.0	1.00	0.93	0.06
**4**	490.5	616.5	591.0	0.95	0.11	0.36
**5**	468.0	582.0	547.5	0.88	0.20	0.57
**6**	640.0		725.0	0.01		>0.01

Let us now discuss the properties of dyes in polar mixtures with a significant water content. The AIE was observed for **4**–**6** for solvent mixtures containing at least 70% of water. The photophysics of the aggregated dye differed compared to the nonaggregated, monomeric molecules in solvents, meaning the bathochromic shift was observed for **1**–**3** (by 15 to 58 nm), while for **4** and **5**, the emission band shifts toward higher energy (by −25 and −35 nm, respectively). One notices that donors of different characters behave differently in the case of FQY change, meaning that ACQ is present in alkyl-based dyes, while AIE prevails in aryl-based donors. Values of τ support our claims regarding AIE/AQC phenomena. If molecules **1**–**3** only exhibited twisted intramolecular charge transfer (TICT) processes without aggregation in polar media, their τ values would be significantly lower than observed ones, which is typical for molecular rotor dyes undergoing TICT, e.g., ThT.^54,55^

We will now discuss the results of 2PA cross section (σ_2_) measurements performed by the two-photon excited fluorescence (2PEF) technique. The two-photon nature of the observed processes was confirmed by the dependence of the 2P fluorescence intensity on the laser power, which is presented in Figure S26. In the case of all dyes, a quadratic dependence was observed, which indicates a two-photon process.

**Figure 3:**
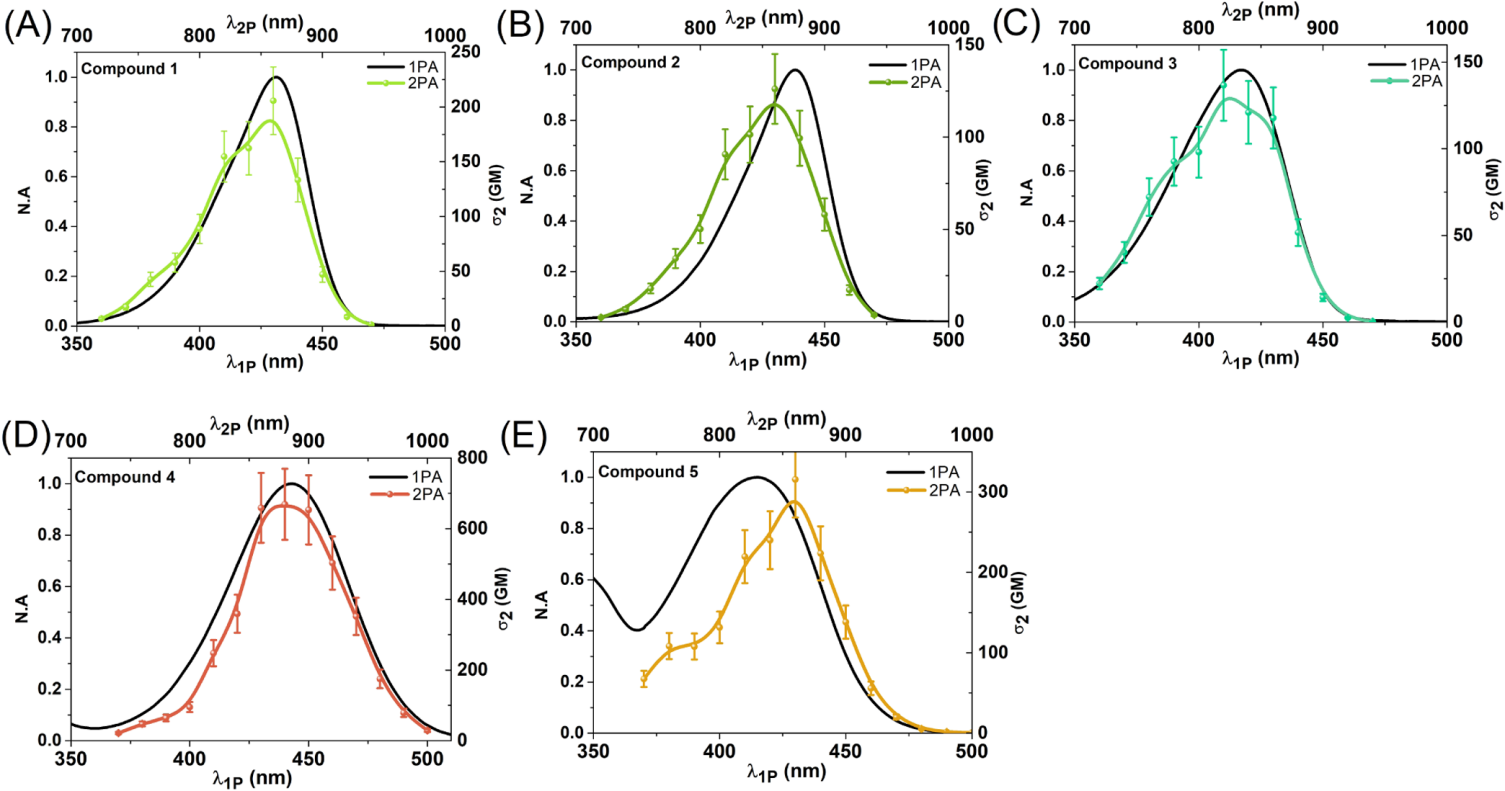
Two-photon absorption (2PA) expressed in σ_2_ and normalized one-photon absorption (1PA) in CHCl_3_. N.A stands for normalized absorbance.

We successfully measured the 2PEF spectra only for dyes **1**–**5**. Dye **6** was not studied due to very weak emission and instrumental limitations. There was no alteration in λ_*em*_ with respect to the type of excitation: one- or two-photon (Figure S27), which indicates transitions from the same electronic state. One- and two-photon absorption spectra are compared for all molecules in Figure 3. The experimental trend of σ_2_ values shows that dyes with aryl-based donors are characterized by values 2–3 times higher than their alkyl counterparts (Figure 3). An opposite trend can be observed for molar absorption coefficients in one-photon-based data (Table 1). Let us now compare the pairs of molecules functionalized with aromatic and nonaromatic donors. For dyes **1** and **4** (−NMe_2_, –NPh_2_), 2PA absorption bands plotted at half the wavelength perfectly match 1PA bands—there are no changes in the peak maxima positions (Figure 3A,D). The next pair of dyes, **2** and **5** (pyrrolidine and carbazol), show small alterations between the 2PA and 1PA spectra (Figure 3B,E). For dye **2**, a slight blue shift was observed in the 2PA band compared to the 1PA band, while aryl derivative **5** shows a red shift in the 2PA band. There are also visible similarities in the 2PA spectra shape for both dyes between 800 and 1000 nm. Molecule **3** presents maxima in 1PA and 2PA spectra in close proximity, and the shape of the 2PA band reveals a vibronic structure. Within the subseries **1**–**3**, the highest experimental σ_2_ was found for the −NMe_2_ donor, while in the case of donors carrying aromatic groups, it was the *N,N*-diphenylamine substituent (AIE active dye characterized by the highest values of σ_2_). Thus, the incorporation of the −NMe_2_/–NPh_2_ pair is the most effective way to obtain efficient two-photon absorbers compared to other investigated donating groups. As far as the potential application of dyes is concerned, let us note that all molecules present two-photon brightness (σ_2,*B*_) well above 50 GM,^7^ which makes them potential fluorescent probes for 2PM (see Figure S28). The best performance in the series is found for **4** with σ_2,*B*_ exceeding 400 GM, while the other dyes present values of σ_2,*B*_ between 120 and 190 GM at band maxima.

Let us now turn to the in-depth analysis of structure–property relationships based on the results of electronic structure calculations. The summary of these calculations is presented in Table 3.

**Table 3:** Calculated 1PA and 2PA Wavelengths (λ [nm]), 1PA Oscillator Strengths, Ground- and Excited-State Dipole Moments (μ [D]), 2PA Strengths (δ^2PA^ [au]), and 2PA Cross Sections (σ_2_ [GM])^a^

comp.	λ^1PA^	oscillator strength	μ (S_0_)	μ (S_1_)	Δμ [*D*]	λ^2PA^	δ^2PA^	σ_2_ [GM]
**1**	385.7	1.45	9.8	18.5	8.7	771.4	44,767	359
**2**	385.0	1.57	10.5	19.0	8.5	770.0	45,708	388
**3**	380.0	1.51	7.8	17.0	9.2	750.0	49,997	307
**4**	412.0	1.49	8.7	21.4	12.7	824.0	110,380	594
**5**	400.4	0.96	4.4	23.2	18.8	800.8	117,052	500

a For dyes **1**–**5**, experimental broadenings were used. Calculations were performed for dyes in chloroform solution.

**Figure 4:**
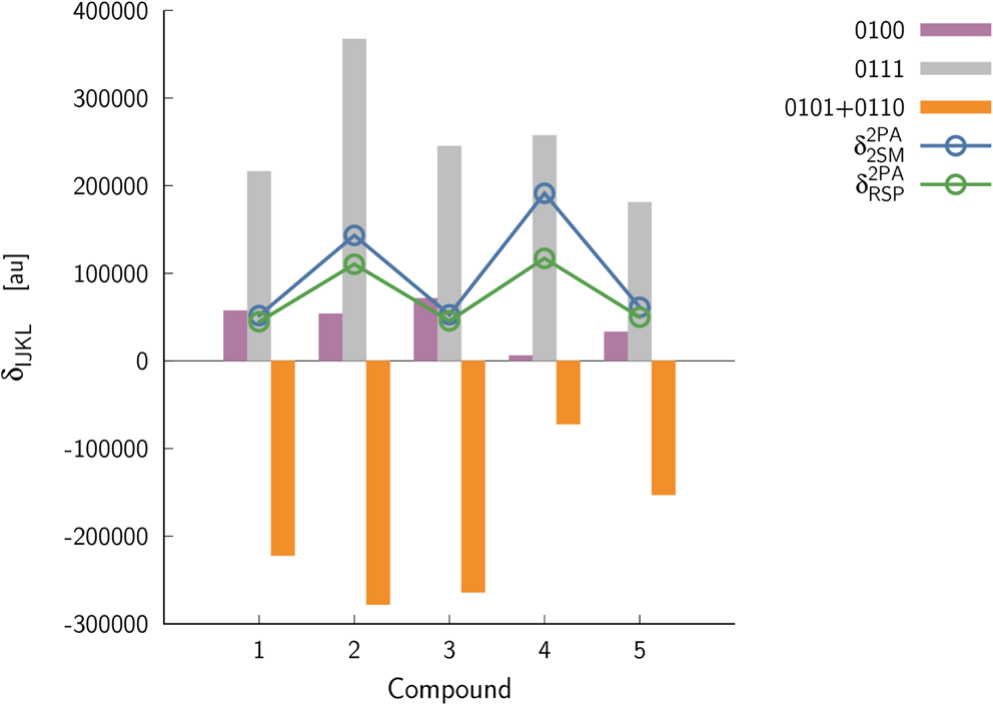
Decomposition of two-photon transition strength under a two-state model (2SM) framework. δ_RSP_^2PA^ corresponds to the response theory value (see Table 3).

In more detail, we determined the two-photon absorption strengths (δ^2PA^, purely molecular parameter dependent on the electronic structure of a molecule) and the two-photon absorption cross section (σ_2_, quantity that can be determined experimentally). The latter parameter given in Table 3 was determined for the maximum of the absorption band represented by a Gaussian-type shape. In so doing, we used experimental values of broadenings for molecules **1**–**5**. All dyes can be grouped in two subsets based on the values of two-photon S_0_ → S_1_ transition strengths: **1**, **2**, and **3** exhibit smaller 2PA activity (307–388 GM), while **4** and **5** exhibit much larger 2PA activity (500–594 GM). In order to understand 2PA activity, we linked it with the electronic structure parameters of each subset. The theoretical framework is given by perturbation theory, which defines δ^2PA^ through sum over transition dipole moments coupling all electronic excited states and their energies. All molecules studied in the present work belong to “dipolar” donor-π-acceptor chromophores. It is well known that the two-photon absorption activity of dipolar dyes can be described with a good approximation by a two-state model involving the ground electronic state and the low-lying intramolecular charge transfer (ICT) state. The spectral feature of the ICT state is not only a large value of wavelength (400–600 nm, or even larger) but also a significant value of the molar absorption coefficient (or oscillator strength). The ICT process also involves a large change in dipole moment between the state in question and the ground state. All these features are present for molecules **1**–**5** in Table 3. In more detail, the absorption wavelength corresponding to the ICT state is located in the range of 380 (**3**)–412 nm (**4**; note that the applied simulation protocol underestimates this value as compared to experimental results); the oscillator strength value is 0.96 (**5**)–1.57 (**2**); and the dipole moment difference upon excitation is as large as 18.8 D (dye **5**; the smallest value equal to 8.5 D is found for dye **2**). The summary of the calculations of the two-state model is shown in Figure 4.

Let us highlight that, except for molecule **5**, the two-state model works very well, as it delivers a value of two-photon transition strength very close to the reference response theory value (all electronic states included in the model). In the case of molecule **5**, the two-state model predicts 191 × 10^3^ au, which is much larger than the response theory value (117 × 10^3^ au). However, for the molecule in question, the three-state model, with the S_2_ state as an intermediate, predicts a 2PA transition strength value much closer to the reference one, i.e., 131 × 10^3^ au. Table 4 contains terms contributing to two-photon transition strength under the three-state model.

**Table 4:** Terms Contributing to the Two-Photon Transition Strength of Molecule **5** under the Three-State Model Framework^a^

IJKL	δ_IJKL_^3SM^
0100	6.2 × 10^3^
0110	–36.2 × 10^3^
0120	4.9 × 10^3^
0101	–36.2 × 10^3^
0111	257.4 × 10^3^
0121	–37.9 × 10^3^
0102	4.9 × 10^3^
0112	–37.9 × 10^3^
0122	5.8 × 10^3^
total	131.0 × 10^3^

a Excited-state S_2_ was taken as an intermediate state.

We find that including additional S_1_/S_2_ couplings (through transition dipole moment between these states) improves the prediction of two-photon transition strength. Figure 4 demonstrates that the final 2PA activity is an interplay of δ_0101_^2PA^ (term involving transition dipole moments between S_0_ and S_1_) and δ_0111_^2PA^ (involving additional dipole moment in the S_1_ state). In the case of two molecules with the largest two-photon activity (**4**, **5**), σ_0111_^2PA^ term is by far the largest, governing the overall response. Upon comparing Δμ (Table 3) with the δ_IJKL_^2PA^ terms (Figure 4), we may identify that the smallest 2PA activity of molecules **1**, **2**, and **3** can be linked with the smallest Δμ values (not exceeding 10 D). These electronic structure parameters can be, on the other hand, related to the efficiency of electron-accepting/-donating moieties present in the studied dyes.

## Conclusions

In this work, we synthesized a novel series of fluorescent dyes based on the benzothiazole core substituted with a palette of donor groups that differ in size, shape, and character (alkyl vs aromatic). The synthesis was followed by detailed spectroscopic characterization (one- and two-photon-excited absorption/emission). The dyes exhibit an intramolecular charge transfer state in their electronic structure. We observed similar photophysical properties in the one- and two-photon regimes for dyes substituted with amine-based, alkyl-functionalized donors, while the aryl-based ones were more diverse in terms of optical properties. We also studied aggregation effects in solvents and mixtures of solvents differing in polarity. The primary finding is that the main difference between alkyl/aryl donors can be linked with the opposite ACQ/AIE mechanisms, i.e., in the water environment, dyes with alkyl donors exhibit ACQ, whereas those with aryl donors demonstrate AIE. Since the electron-accepting core remains intact, the substituent-dependent AIE/ACQ properties might have interesting implications in the case of dyes interacting with hydrophobic pockets in biomolecules. Since both ACQ and AIE can be used in the detection of biomolecules, our studies are beneficial for a range of fluorescent-detected applications. Moreover, multiphoton absorption studies were performed to determine the potential of the dyes in two-photon microscopy. The results of spectroscopic multiphoton measurements were supported by electronic structure calculations using coupled-cluster theory. It was found that higher σ_2_ for molecules with aromatic substituents can be linked with higher Δ*μ* values in the intramolecular charge transfer excited state. The most promising structures for the two-photon bioimaging are the ones substituted with the −NMe_2_ donor and its aromatic counterpart, −NPh_2_. Overall, the results presented in this study provide an indication regarding the optimal substituents to achieve bright emission in various media and how these properties are related to aggregation effects.

## Supporting Information Available

Detailed synthesis description; NMR spectra and additional photophysical data; spectra and charts used in the analysis of the results (PDF)
