# Supplementary material for: Thioflavin T Inspirations: On the Photophysical and Aggregation Properties of Fluorescent Difluoroborates Based on the Benzothiazole Core
Source: J Phys Chem A. 2025 Apr 9;129(16):3663–71. doi: 10.1021/acs.jpca.5c01254 (PMC12040292; doi:10.1021/acs.jpca.5c01254)
Supplement: Supplementary file 1 — jp5c01254_si_001.pdf [file jp5c01254_si_001.pdf]

Supporting Information: Thioflavin T Inspirations: On the Photophysical and Aggregation Properties of Fluorescent Difluoroborates Based on Benzothiazole Core

Patryk Rybczyński,[†,¶] Agata Hajda,[‡,¶] Robert Zaleśny,[*,‡] Borys Ośmiałowski,[*,†] and Joanna Olesiak-Bańska[*,‡]

†*Faculty of Chemistry, Nicolaus Copernicus University, Gagarina Street 7, 87-100 Toruń, Poland*

‡*Faculty of Chemistry, Wroclaw University of Science and Technology, Wybrzeże Wyspiańskiego 27, 50-370 Wrocław, Poland*

¶*These authors contributed equally.*

E-mail: robert.zalesny@pwr.edu.pl; borys.osmialowski@umk.pl; joanna.olesiak-banska@pwr.edu.pl

Contents

1 Synthesis S3

2 NMR spectra S4

3 Additional photophysical data and figures S16

1 Synthesis

Amides were prepared by the reaction between the corresponding acid chlorides (1 eq), in anhydrous THF, with 2-amino-6-(trifluoromethyl)benzothiazole (1eq) in the presence of triethylamine (1 eq). All dyes were obtained by the reaction of amides with BF_3 etherate and DIEA as a base in anhydrous DCM.

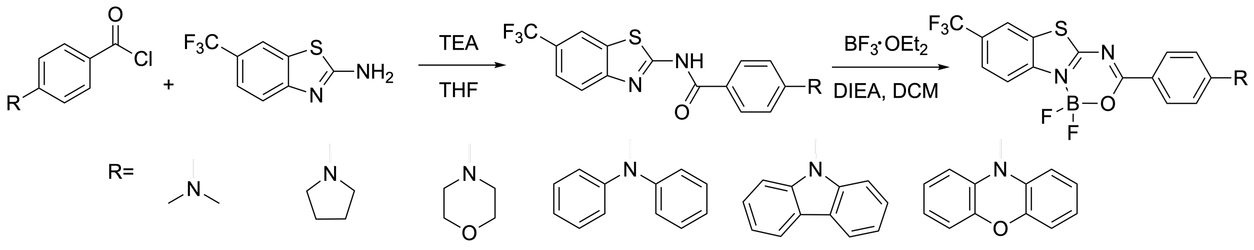

1 2 3 4 5 6

2 NMR spectra

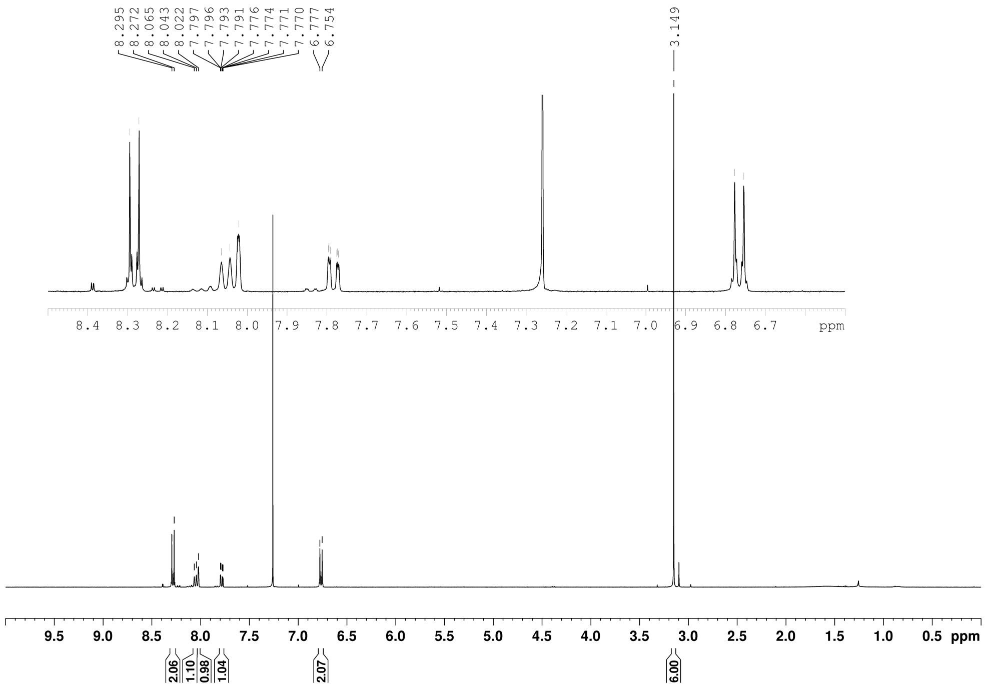

Figure S1: ^{1}H NMR for compound **1**

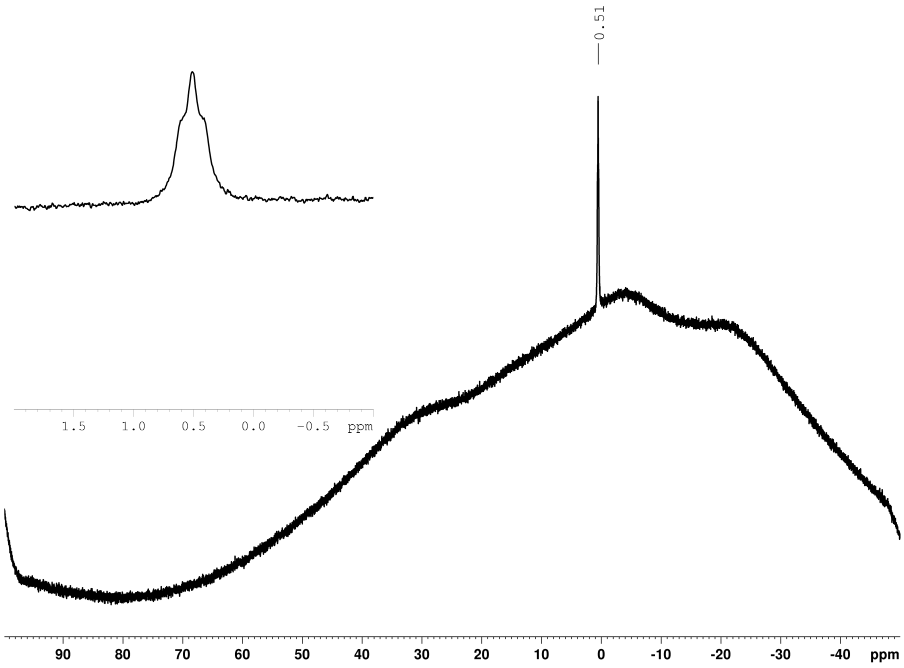

Figure S2: ^{11}B NMR for compound **1**

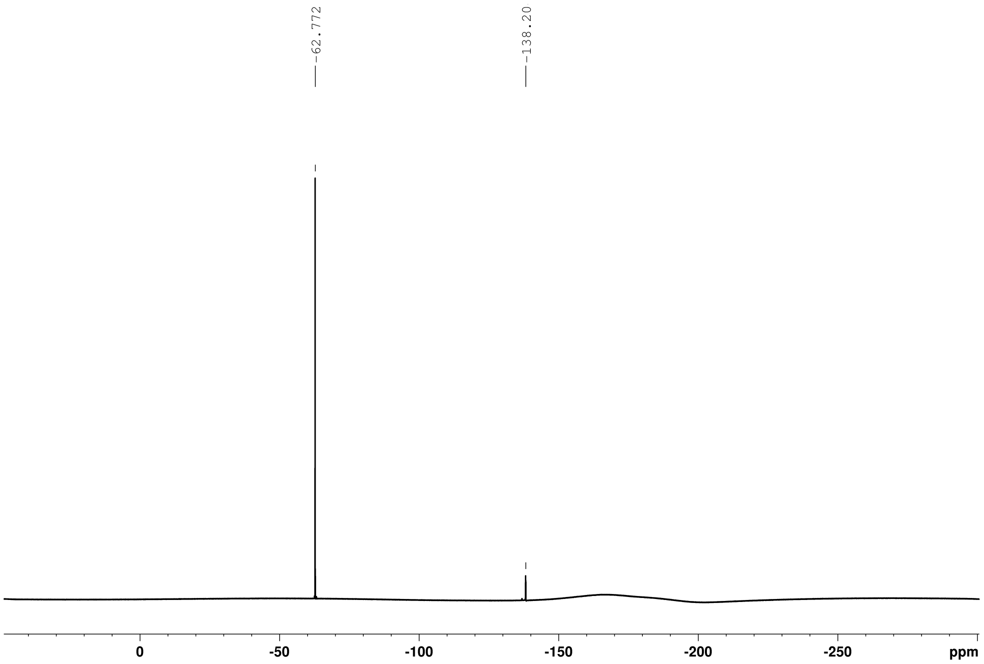

Figure S3: ^{19}F NMR for compound **1**

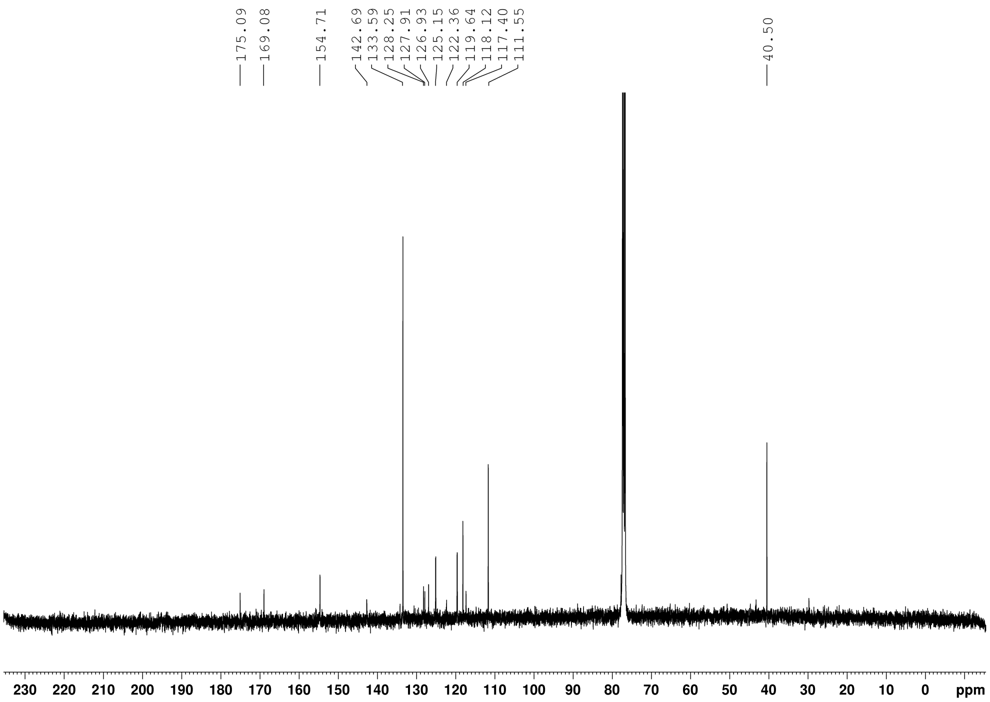

Figure S4: ^{13}C{^{1}H} NMR for compound **1**

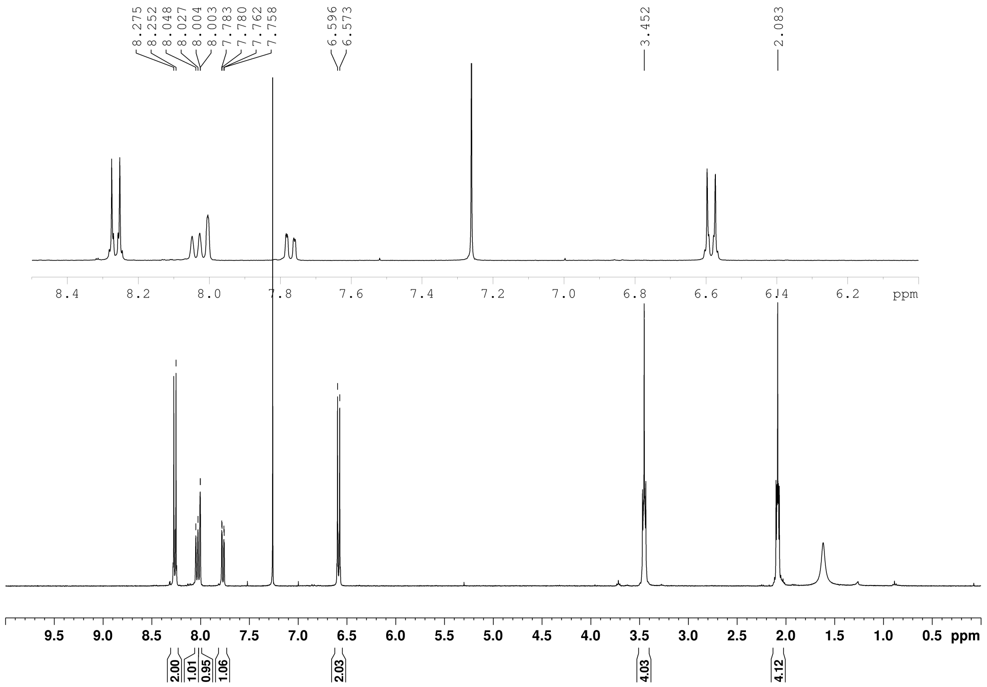

Figure S5: ^{1}H NMR for compound **2**

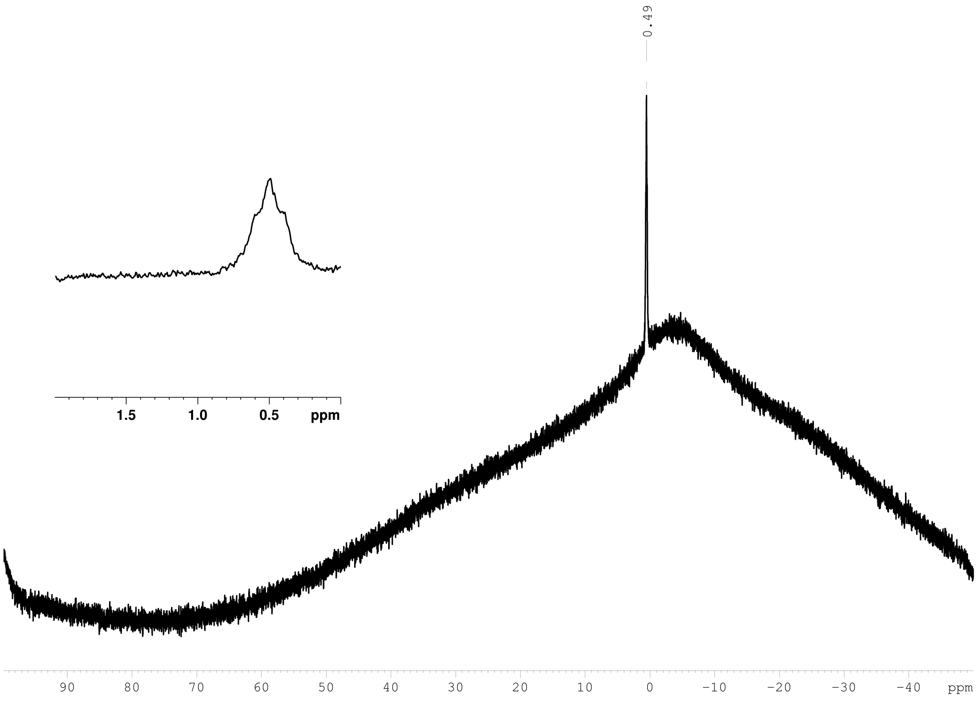

Figure S6: ^{11}B NMR for compound **2**

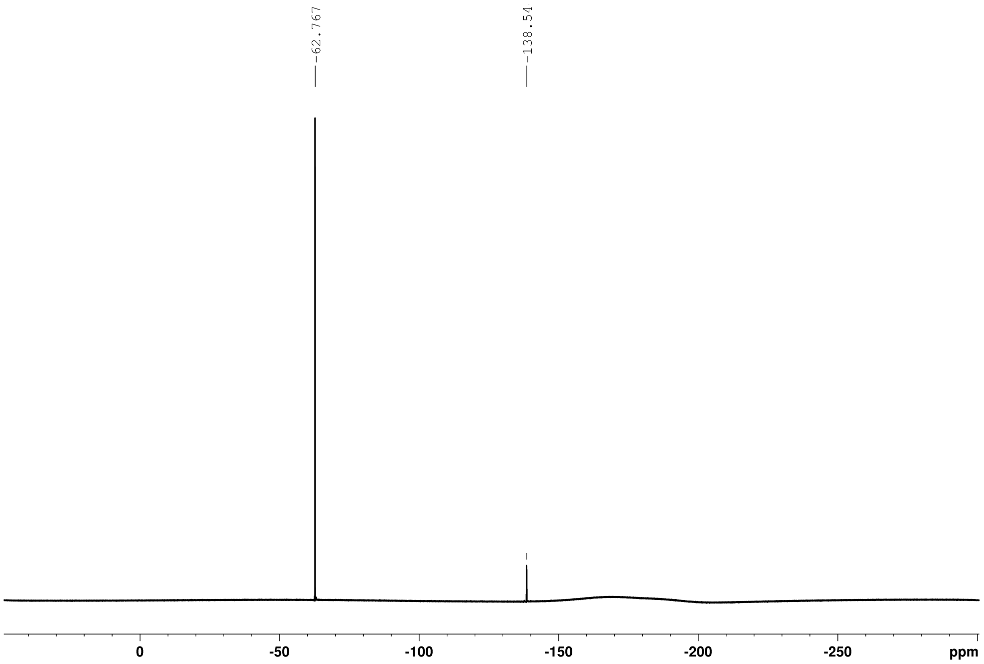

Figure S7: ^{19}F NMR for compound **2**

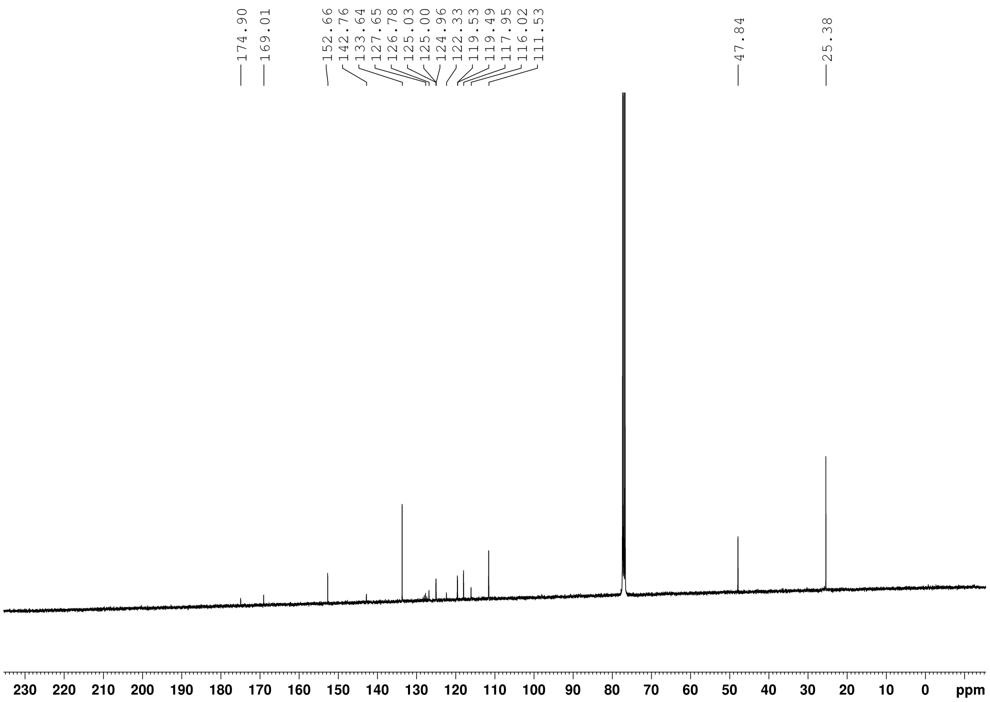

Figure S8: $^{13}C\{^{1}H\}$ NMR for compound **2**

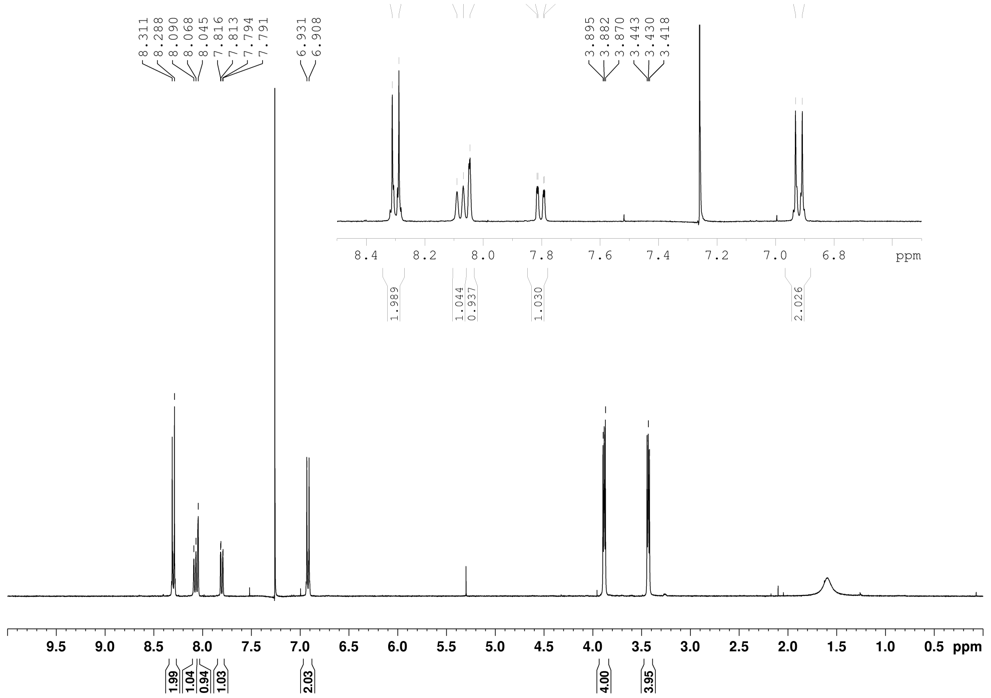

Figure S9: ^{1}H NMR for compound **3**

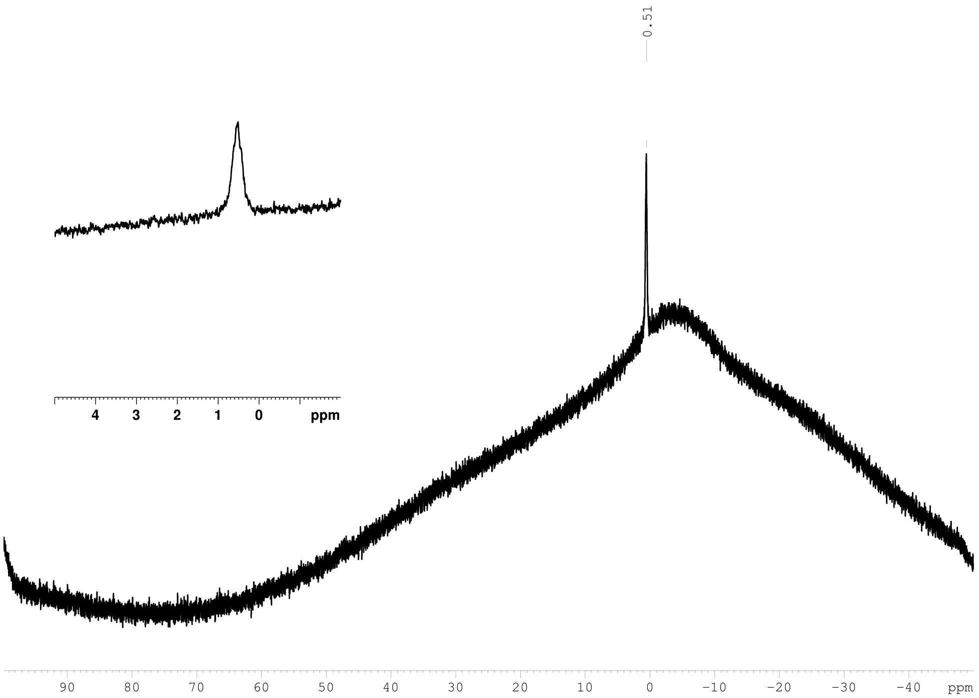

Figure S10: ^{11}B NMR for compound **3**

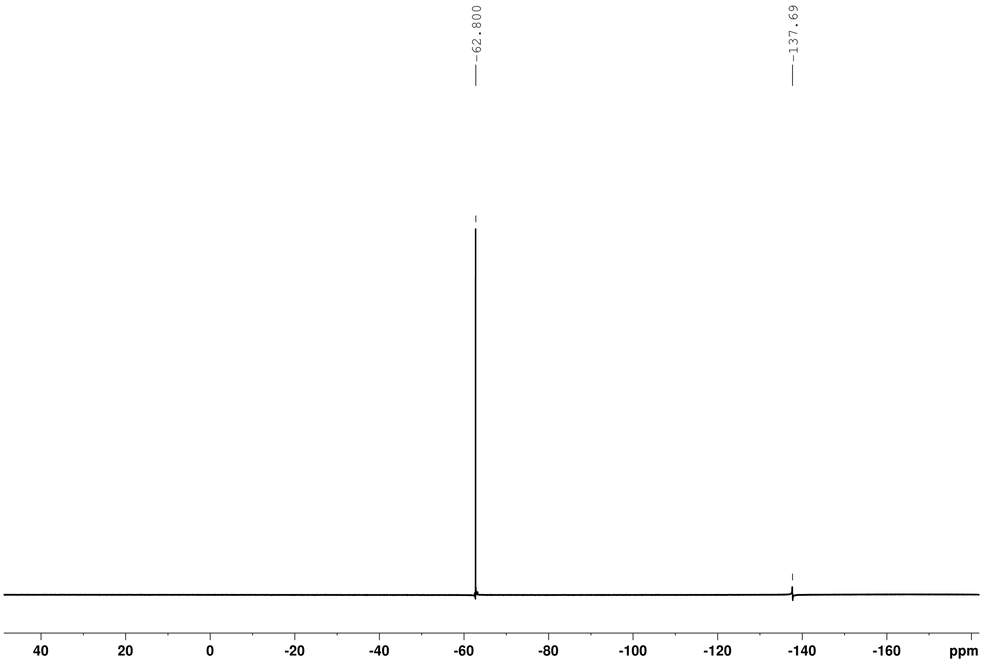

Figure S11: ^{19}F NMR for compound **3**

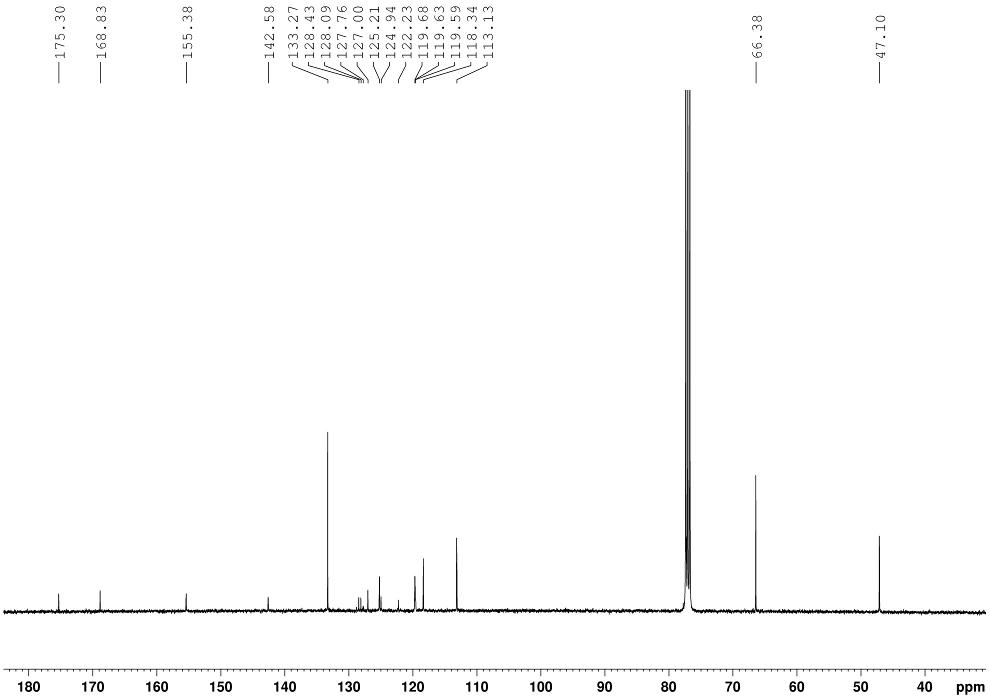

Figure S12: ^{13}C{^{1}H} NMR for compound **3**

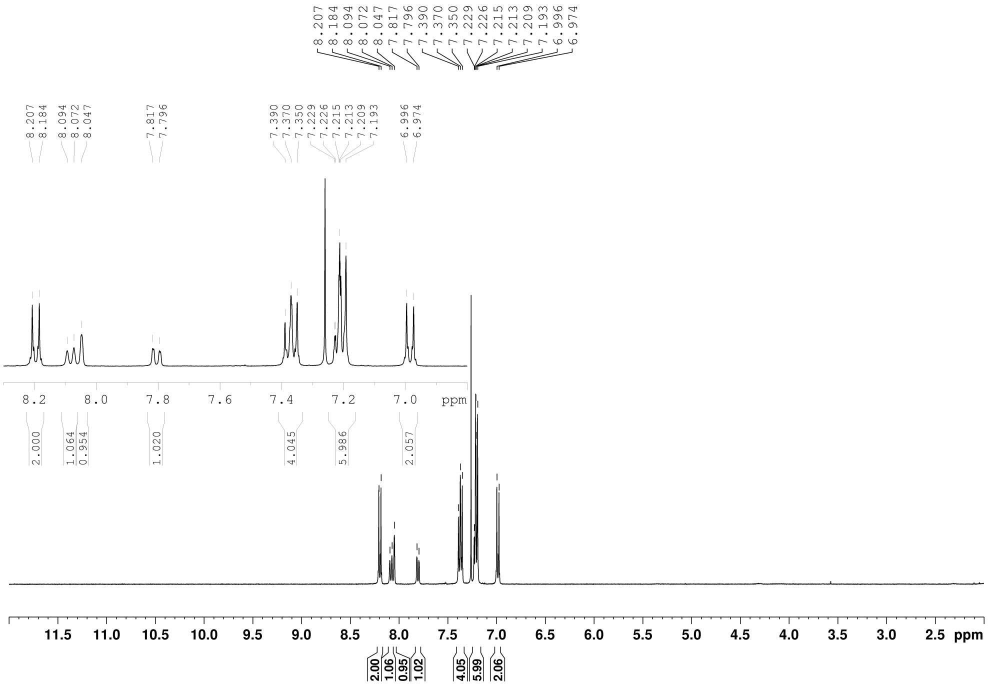

Figure S13: ^{1}H NMR for compound **4**

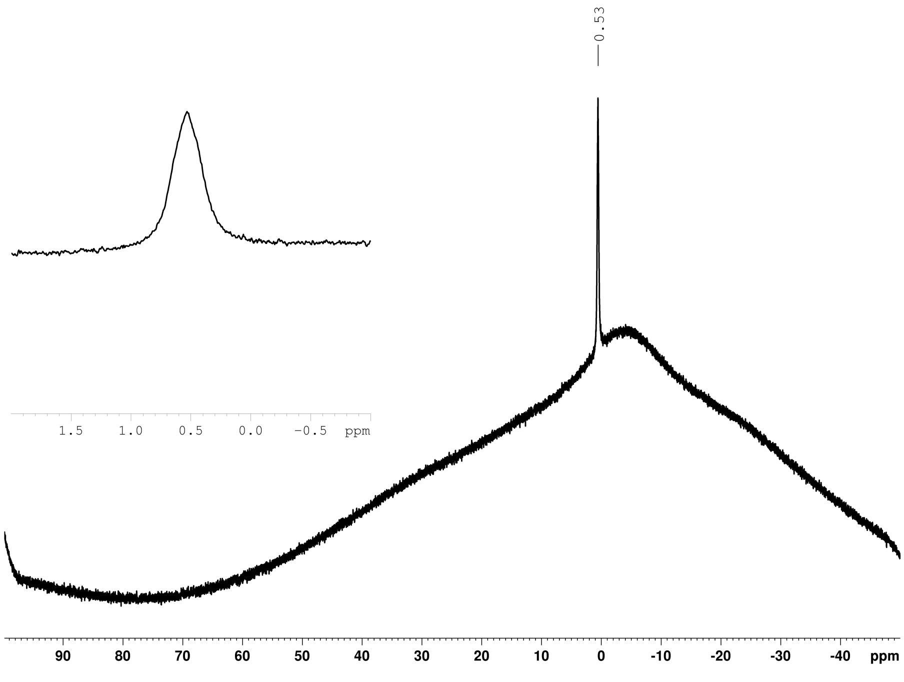

Figure S14: ^{11}B NMR for compound **4**

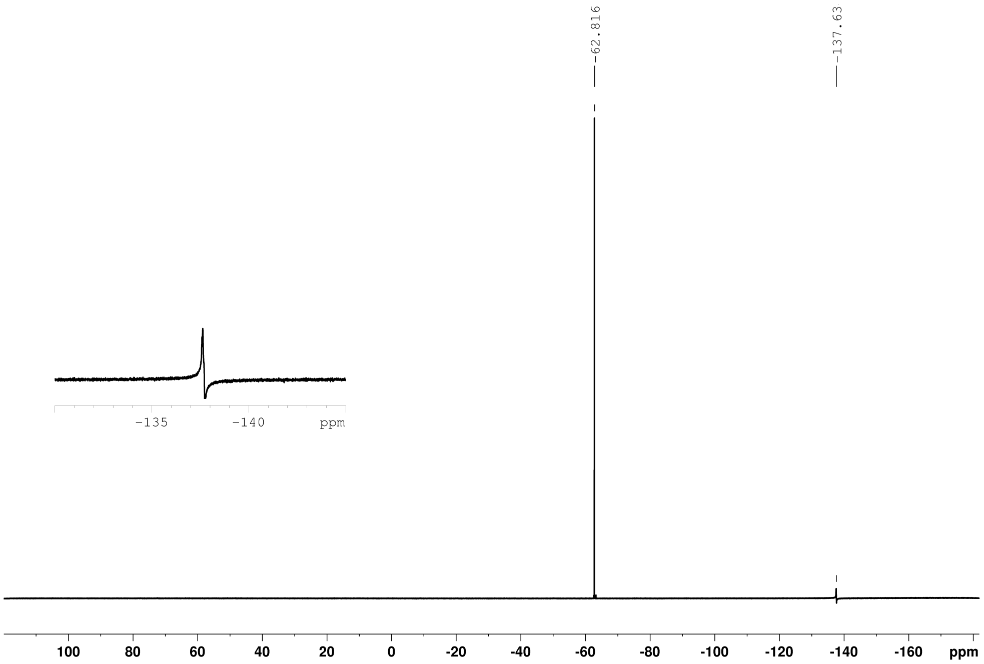

Figure S15: ^{19}F NMR for compound **4**

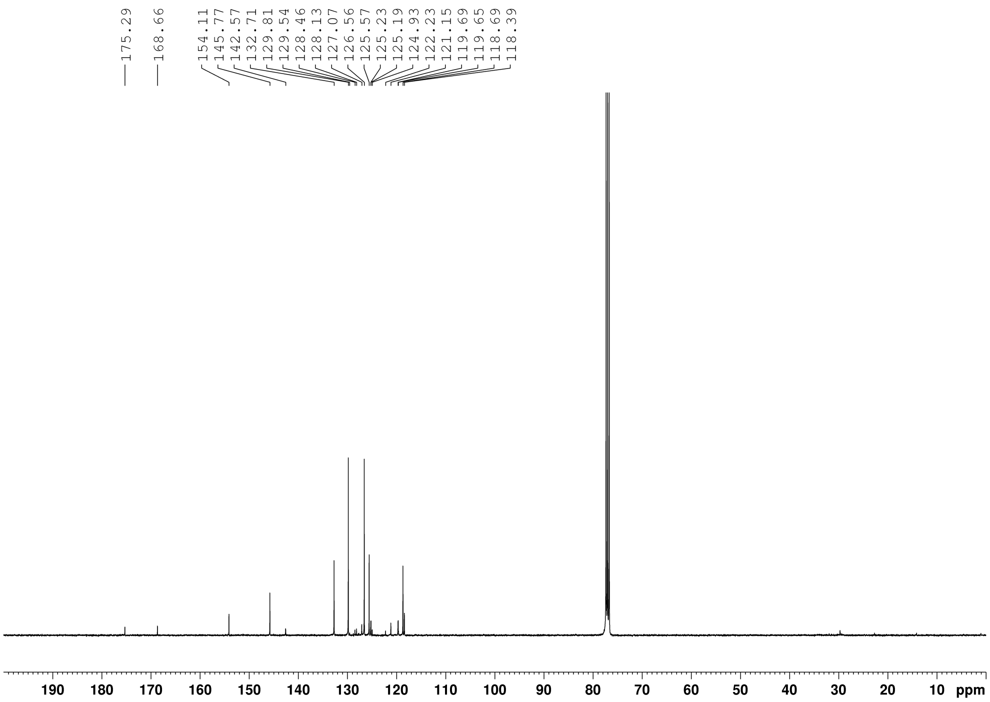

Figure S16: ^{13}C{^{1}H} NMR for compound **4**

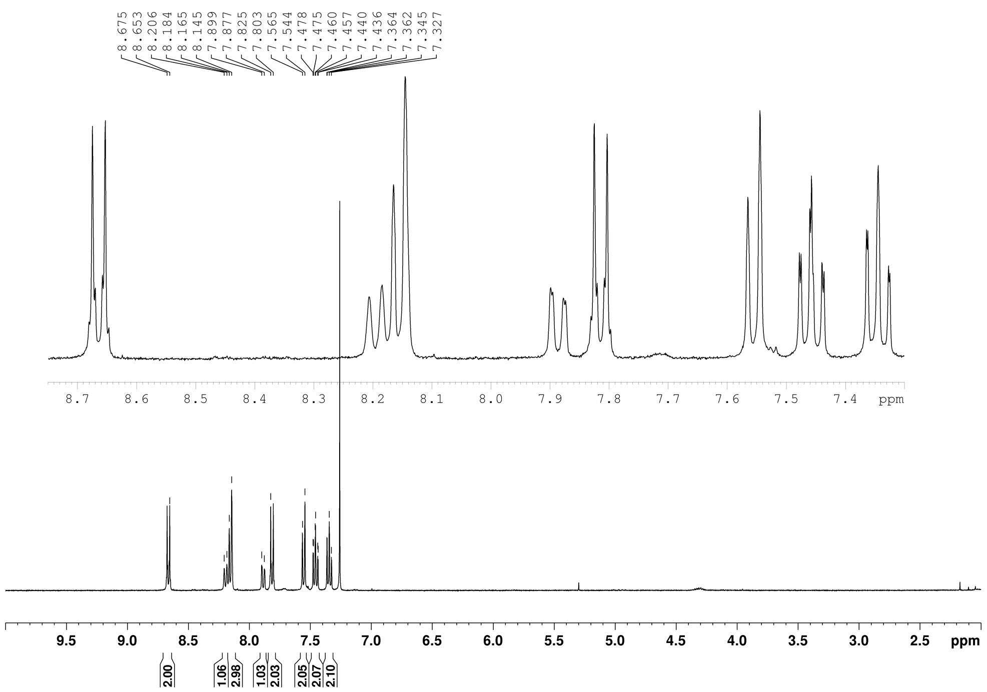

Figure S17: ^{1}H NMR for compound **5**

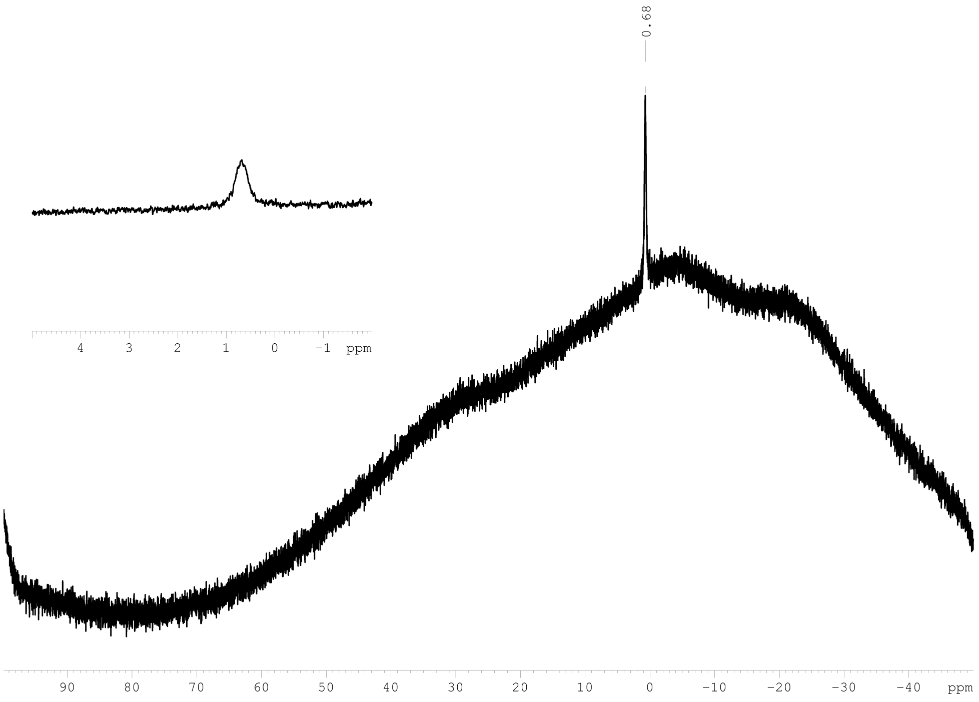

Figure S18: ^{11}B NMR for compound **5**

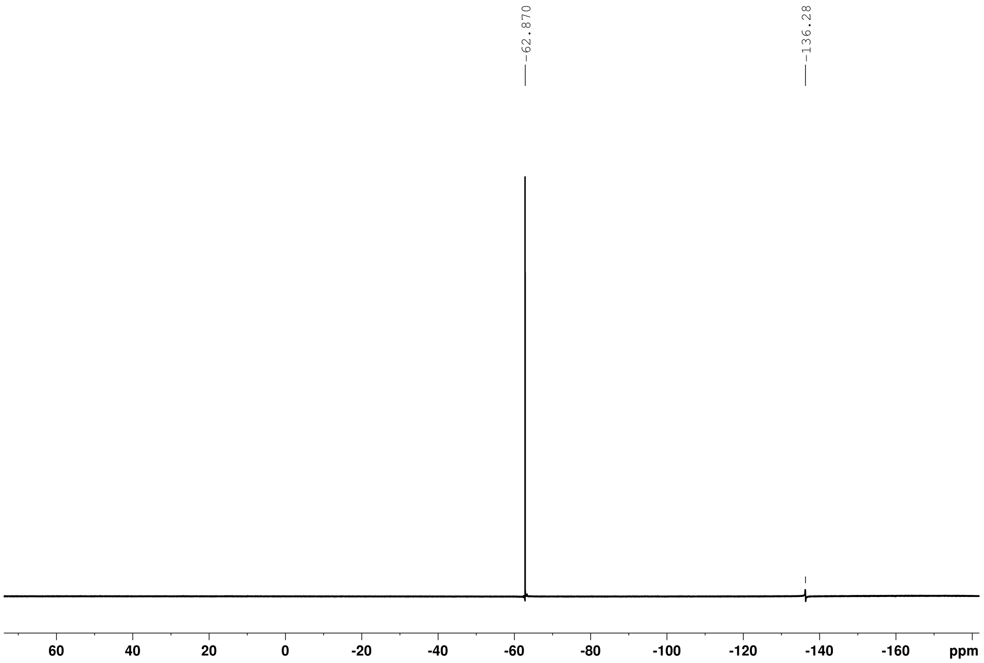

Figure S19: ^{19}F NMR for compound **5**

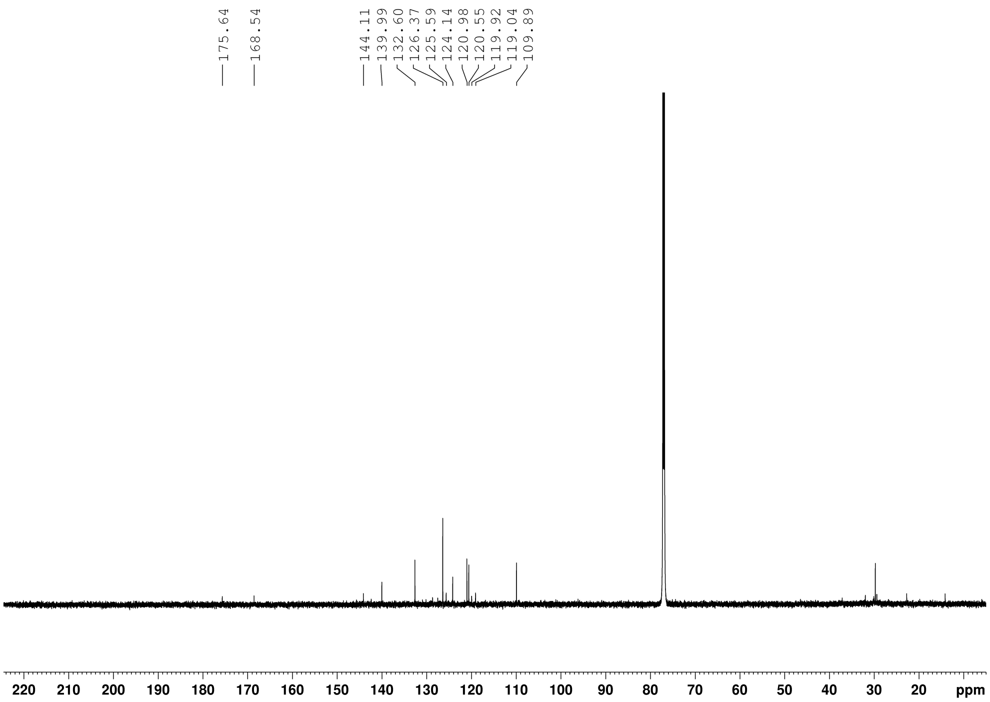

Figure S20: $^{13}C\{^{1}H\}$ NMR for compound **5**

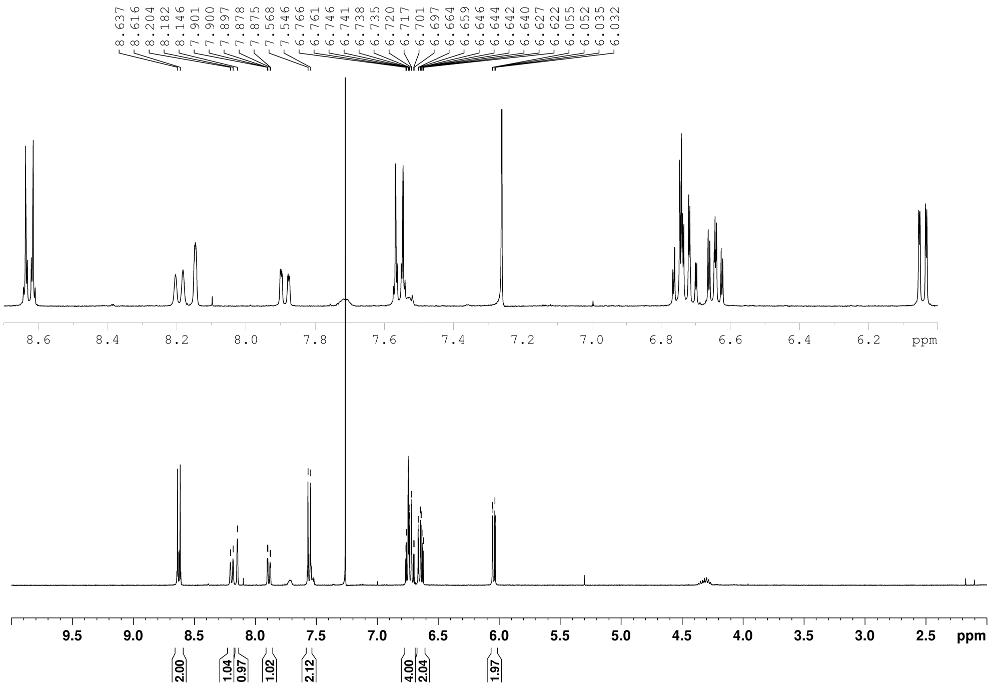

Figure S21: ^{1}H NMR for compound **6**

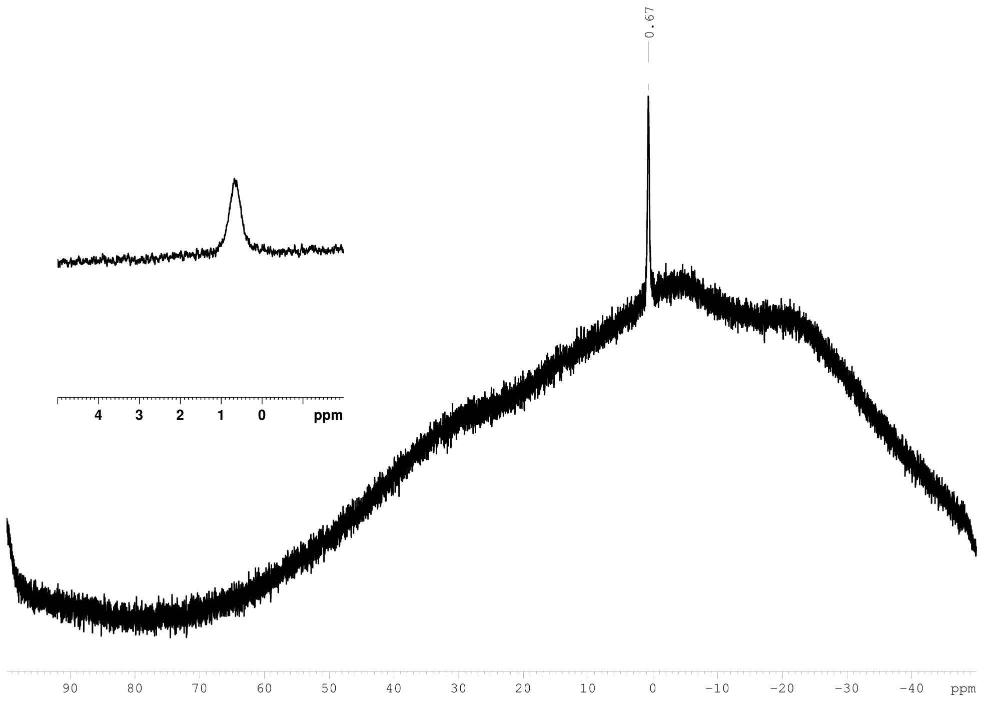

Figure S22: ^{11}B NMR for compound **6**

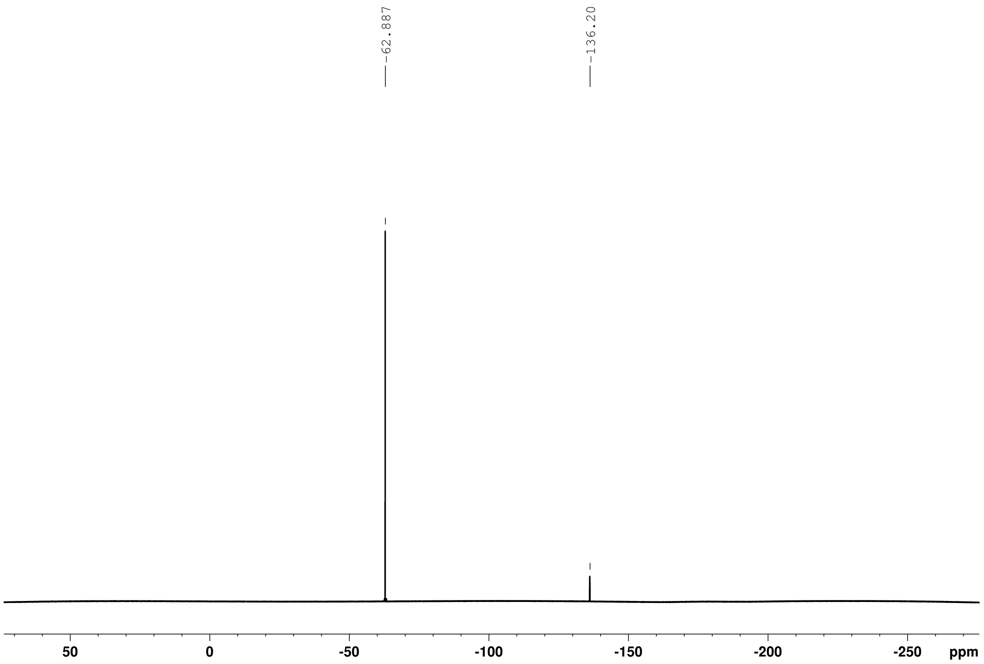

Figure S23: ^{19}F NMR for compound **6**

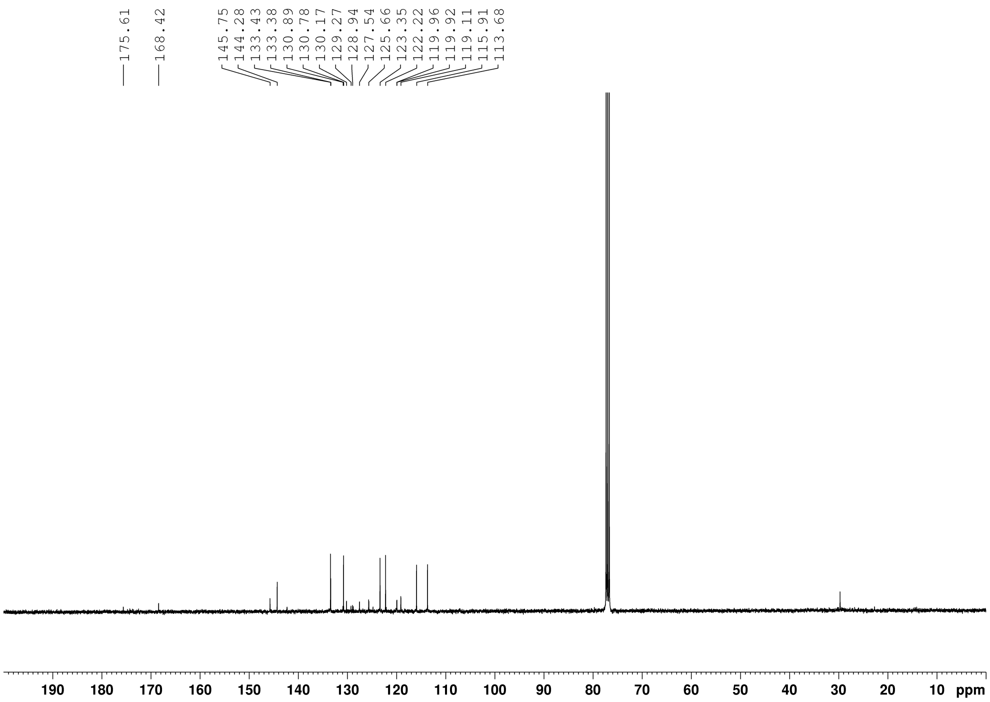

Figure S24: $^{13}C\{^{1}H\}$ NMR for compound **6**

3 Additional photophysical data and figures

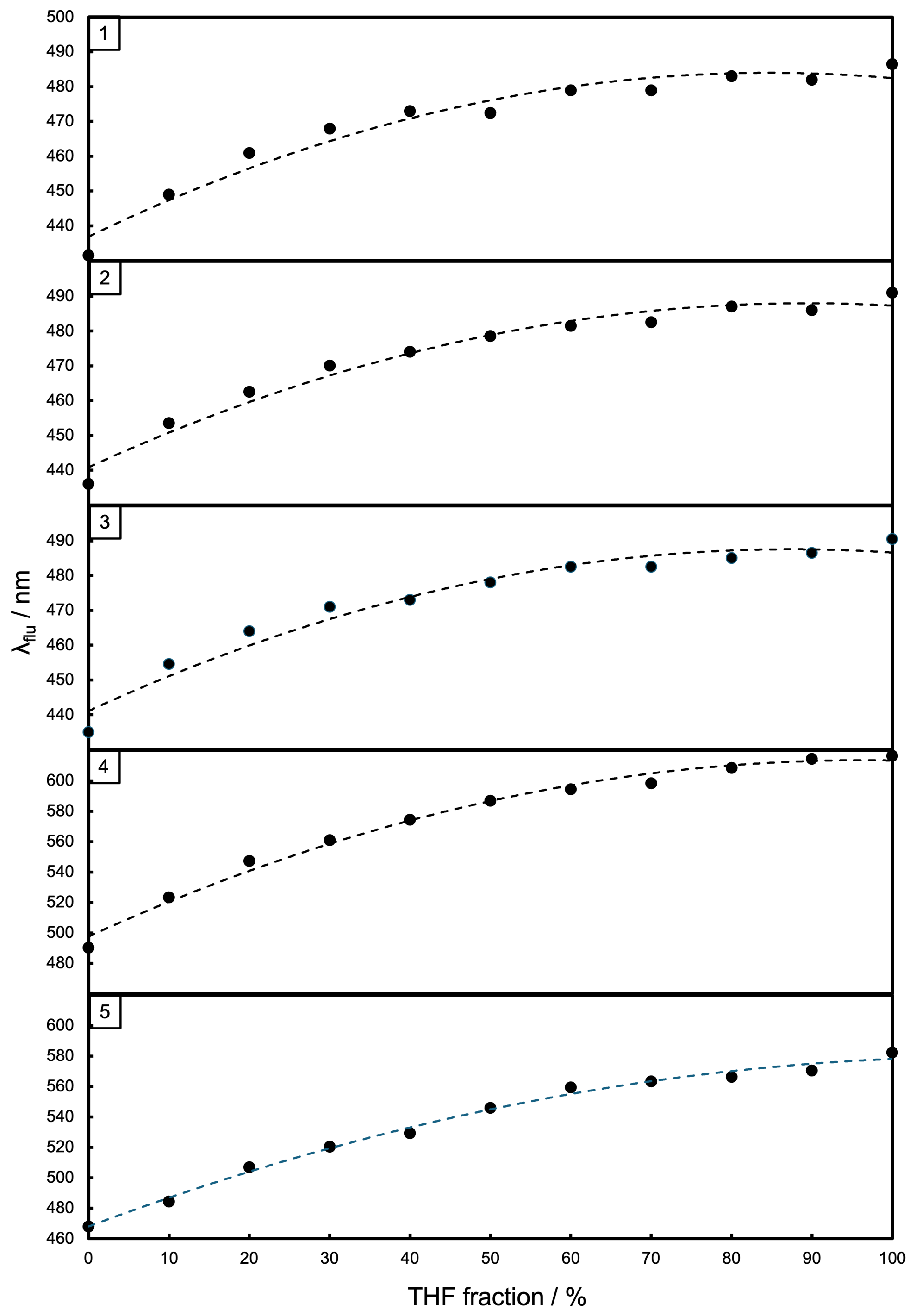

Figure S25: The dependence of the emission maximum on the composition of the hexane/THF mixture.

Table S1: Fluorescence lifetime τ [ns] and radiative k_r and non-radiative k_{nr} transition rate constants [10^9 s^{-1}] determined in hexane, THF, and aggregates (THF/water).

	Hexane			THF			Aggregates		
Compound	τ	k_r	k_{nr}	τ	k_r	k_{nr}	τ	k_r	k_{nr}
1	1.54	0.62	0.03	1.81	0.46	0.09	4.23	0.01	0.23
2	1.53	0.65	0.01	1.98	0.51	0.00	2.62	0.01	0.38
3	1.50	0.67	0.00	1.86	0.50	0.05	1.82	0.03	0.51
4	3.24	0.29	0.01	0.95	0.11	0.94	3.82	0.09	0.17
5	3.50	0.25	0.04	2.61	0.08	0.30	7.72	0.07	0.06
6	0.86	0.02	1.14	-	-	-	-	-	-

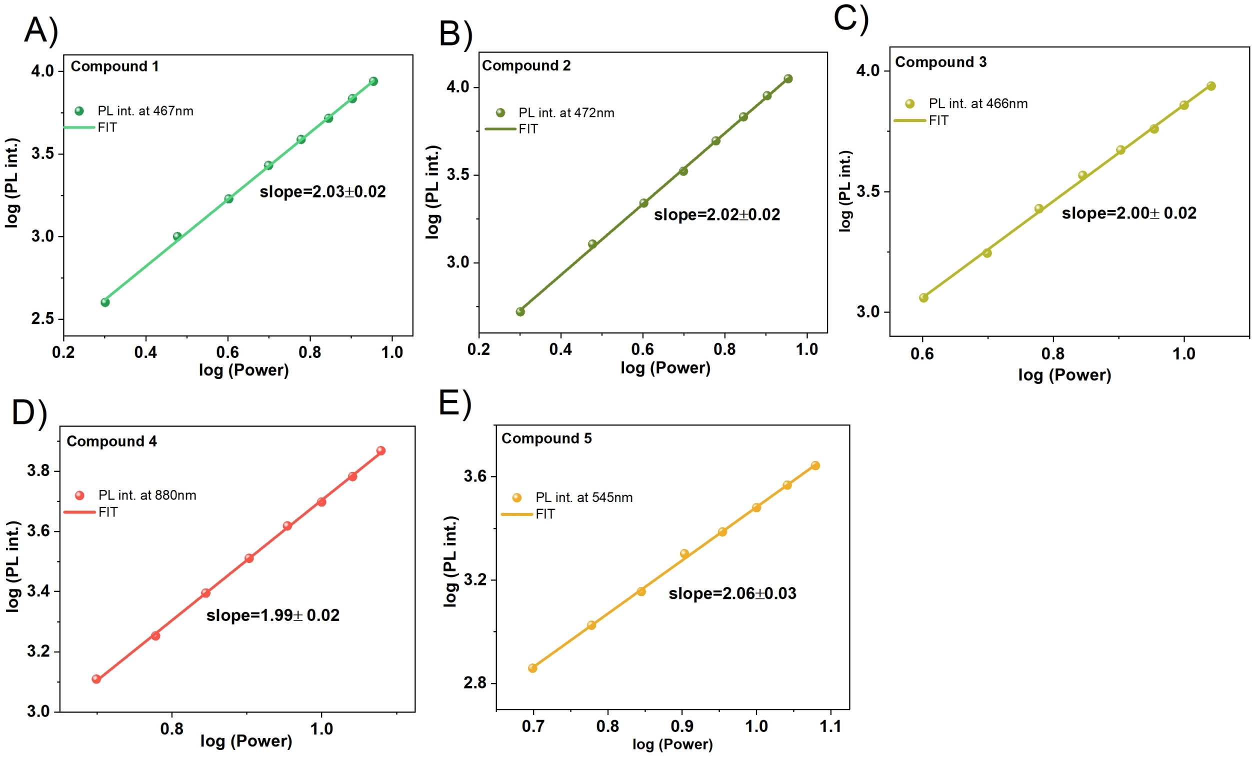

Figure S26: Log-log plots of the photoluminescence (PL) intensity dependence on laser power. Slopes indicate power exponent (n).

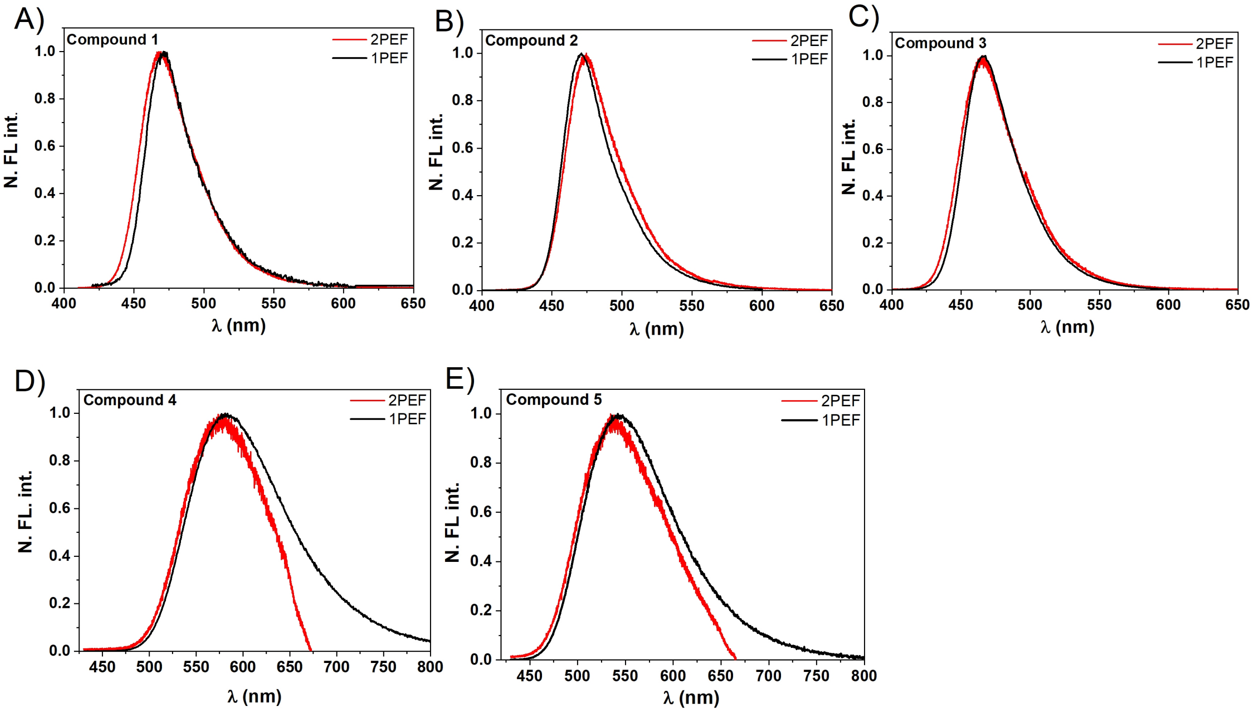

Figure S27: Comparison of one-photon excited fluorescence (1PEF) and two-photon excited fluorescence (2PEF) for all measured compounds.

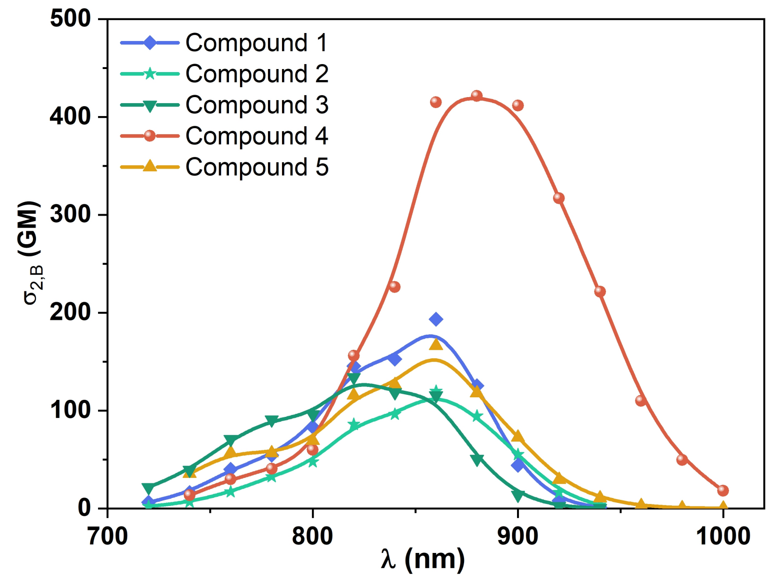

Figure S28: Comparison of two-photon brightness ($\sigma_{2,B}$) for all measured compounds. Error value is 15%.

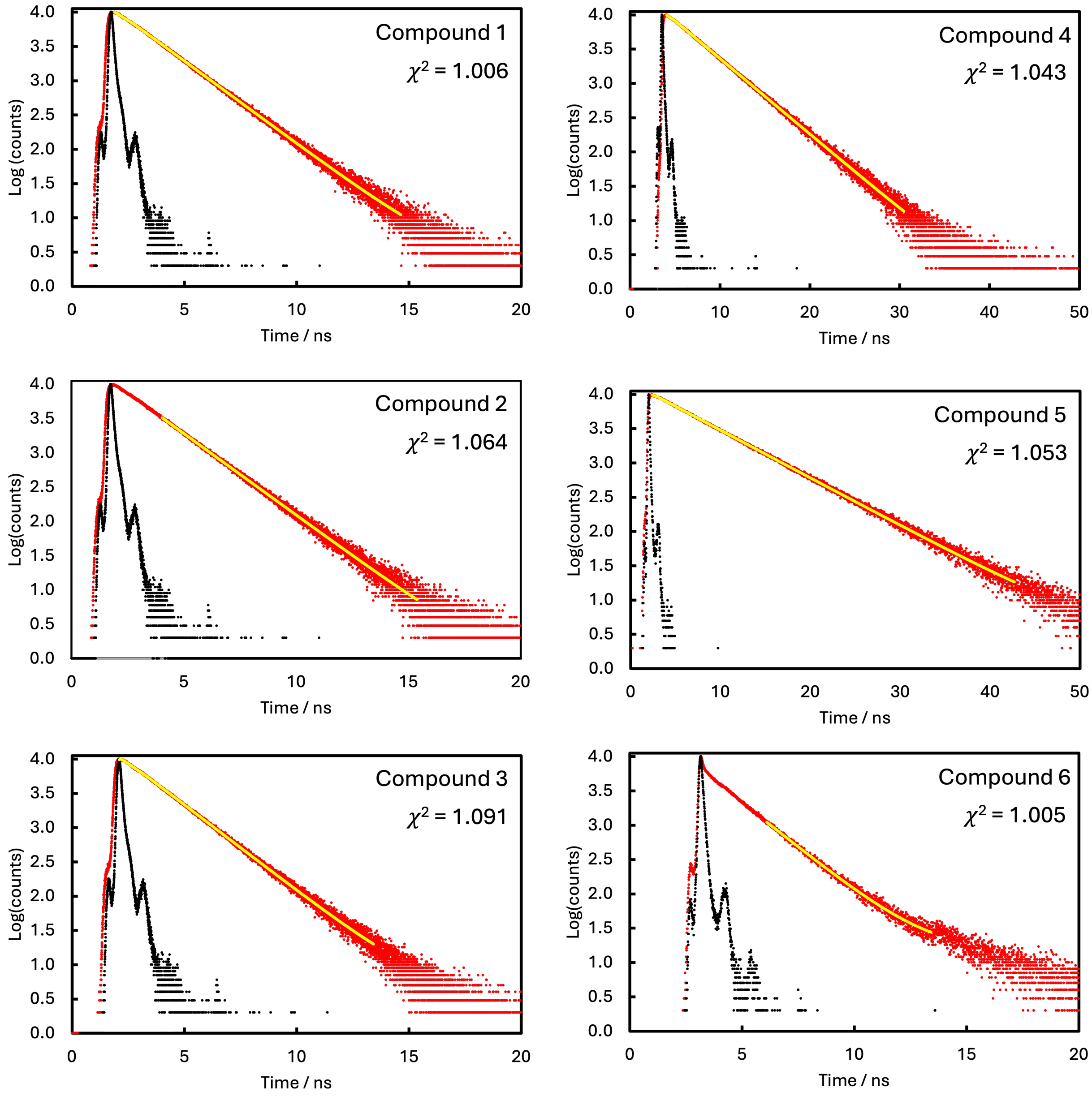

Figure S29: The fluorescence decays (red), IRF (black) and fitted curve (yellow) and obtained values of χ^2 for compounds **1-6** in chloroform

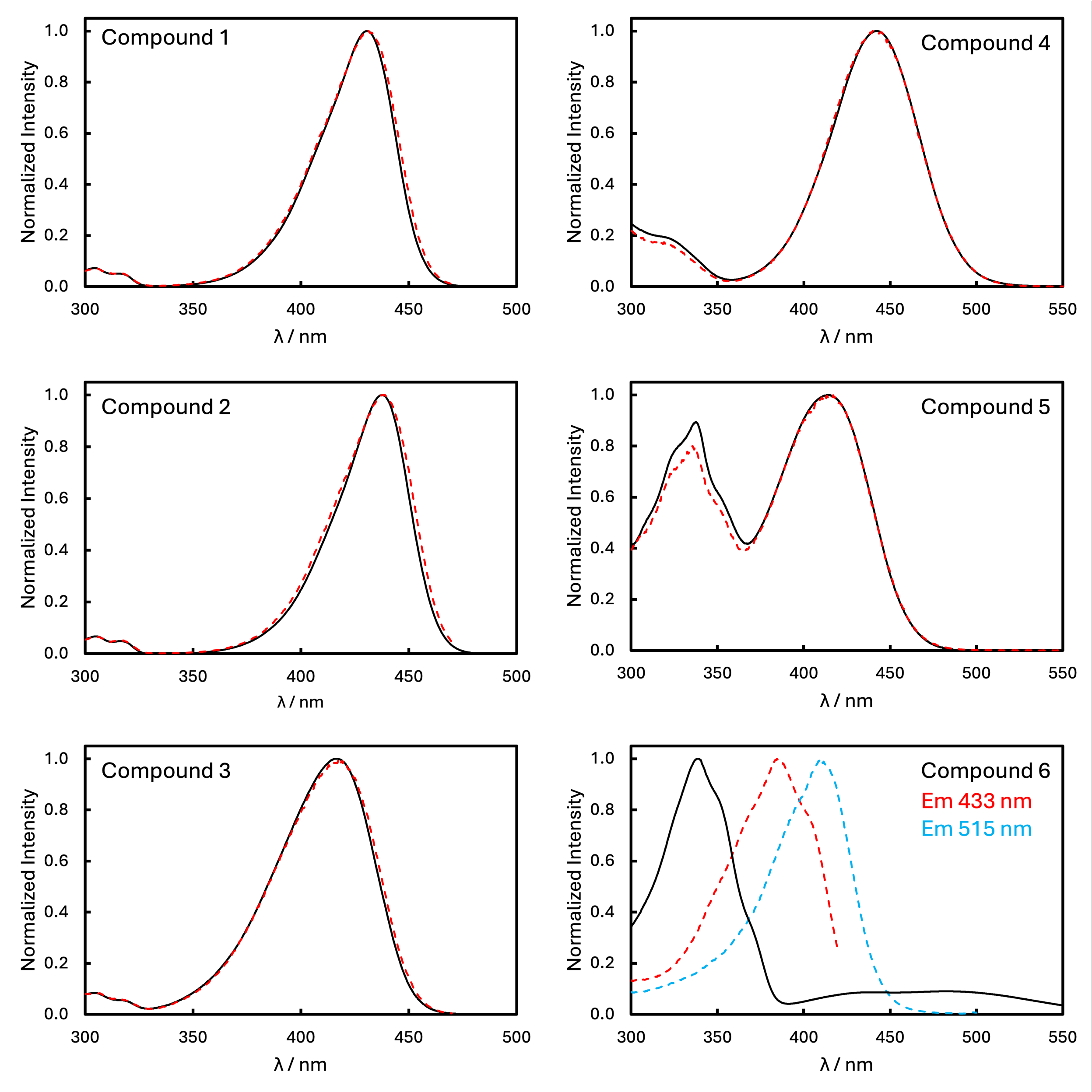

Figure S30: The absorption (solid red line) and excitation (dashed line) spectra for compounds **1-6** in chloroform
